# Plasma MicroRNA Profiling of *Plasmodium falciparum* Biomass and Association with Severity of Malaria Disease

**DOI:** 10.3201/eid2702.191795

**Published:** 2021-02

**Authors:** Himanshu Gupta, Mercedes Rubio, Antonio Sitoe, Rosauro Varo, Pau Cisteró, Lola Madrid, Inocencia Cuamba, Alfons Jimenez, Xavier Martiáñez-Vendrell, Diana Barrios, Lorena Pantano, Allison Brimacombe, Mariona Bustamante, Quique Bassat, Alfredo Mayor

**Affiliations:** ISGlobal, Hospital Clinic—Universitat de Barcelona, Barcelona, Spain (H. Gupta, M. Rubio, R. Varo, P. Cisteró, A. Jimenez, X. Martiáñez-Vendrell, D. Barrios, A. Brimacombe, M. Bustamante, Q. Bassat, A. Mayor);; Centro de Investigação em Saúde de Manhiça (CISM), Maputo, Mozambique (A. Sitoe, R. Varo, L. Madrid, I. Cuamba, Q. Bassat, A. Mayor);; Spanish Consortium for Research in Epidemiology and Public Health (CIBERESP), Madrid, Spain (A. Jimenez, M. Bustamante, Q. Bassat, A. Mayor);; Harvard T.H. Chan School of Public Health Department of Biostatistics, Boston, Massachusetts, USA (L. Pantano);; Universitat Pompeu Fabra (UPF), Barcelona (M. Bustamante); I; nstitut Català de Recerca en Estudis Avançats (ICREA), Barcelona (Q. Bassat);; Hospital Sant Joan de Déu—University of Barcelona Pediatrics Department, Barcelona (Q. Bassat)

**Keywords:** *Plasmodium falciparum*, miRNA, malaria, severe malaria, biomarkers, next-generation sequencing, histidine-rich protein 2, vector-borne infections, Mozambique, parasites, Spain

## Abstract

Severe malaria (SM) is a major public health problem in malaria-endemic countries. Sequestration of *Plasmodium falciparum*–infected erythrocytes in vital organs and the associated inflammation leads to organ dysfunction. MicroRNAs (miRNAs), which are rapidly released from damaged tissues into the host fluids, constitute a promising biomarker for the prognosis of SM. We applied next-generation sequencing to evaluate the differential expression of miRNAs in SM and in uncomplicated malaria (UM. Six miRNAs were associated with in vitro *P. falciparum* cytoadhesion, severity in children, and *P. falciparum* biomass. Relative expression of hsa-miR-4497 quantified by TaqMan-quantitative reverse transcription PCR was higher in plasma of children with SM than those with UM (p<0.048) and again correlated with *P. falciparum* biomass (p = 0.033). These findings suggest that different physiopathological processes in SM and UM lead to differential expression of miRNAs and pave the way for future studies to assess their prognostic value in malaria.

Case-fatality rates for *Plasmodium falciparum* severe malaria (SM) remain unacceptably high in young children in Africa (*1*). Early detection and prompt treatment of SM are critical to improve the prognosis of sick children. Unfortunately, clinical signs and symptoms in many malaria patients, particularly early in the infection, may not adequately indicate whether the infection will trigger severe or life-threatening disease. Moreover, in malaria-endemic areas, where immunity to malaria is progressively acquired, detecting peripheral *P. falciparum* parasitemia in sick children does not necessarily prove that malaria is the cause of the severe pathology observed, given that many persons may carry parasites without expressing clinical malarial disease (*2*).

Sequestration of *P. falciparum*–infected erythrocytes (iEs) (*3*) in vital organs is considered a key pathogenic event leading to SM, as has been shown in postmortem parasite counts in patients who died with cerebral malaria (*4**,**5*). This extensive sequestration of parasitized erythrocytes in the microvasculature, together with the production of inflammatory mediators, leads to the dysfunction of one or more peripheral organs, such as the lungs (acute respiratory distress syndrome), kidneys (acute kidney injury) or brain (coma) (*6**,**7*). This tissue-specific tropism of *P. falciparum* parasites is mediated by the *P. falciparum* erythrocyte membrane protein 1 (PfEMP1), which can bind to different host receptors on the capillary endothelium, uninfected erythrocytes, and platelets (*8**,**9*); such receptors include endothelial receptor of protein C (ePCR), gC1qR, intercellular adhesion molecule-1, CD36, chondroitin sulfate A, or complement receptor 1 (*10*).

Efforts have been made to identify biomarkers of SM that could be used for early diagnosis and for reducing severity of disease (*11*). Several biomarkers related to endothelial activation and immune dysfunction have been associated with different malaria-derived severe pathologies (*11**–**14*). Plasma level of histidine-rich protein 2 (HRP2), a parasite-specific protein secreted by the parasite during its blood cycle, has been used as a biomarker of total parasite biomass (circulating and sequestered parasites) (*15**,**16*) and therefore as a prognostic marker of the total parasite biomass and as a better proxy marker for SM than peripheral parasitemia (*16*). Organ damage and pathological disease states have also been associated with the rapid release of microRNAs (miRNAs), a class of endogenous small noncoding RNAs (18–24 nt), into circulation (*17*). Because secreted miRNAs can be detected in biologic fluids such as plasma (*18*), they are currently being explored (*17*) as promising noninvasive biomarkers to monitor organ functionality and tissue pathophysiological status. The content of miRNAs in the host is influenced by host-pathogen interactions (*19*). Sequestration of erythrocytes infected with *P. berghei* in mice brains has been demonstrated to modify the miRNA expression in cells (*20*). Similarly, sequestration of *P. vivax* gametocytes in bone marrow has been associated with transcriptional changes of miRNAs involved in erythropoiesis (*21*). The evidence suggests that *Plasmodium* parasites, although unable to produce miRNAs (*22*), could affect the production of organ-specific host miRNAs, pointing toward the potential of these small molecules to detect SM associated organ injury (*23*) and to confirm the contribution of malaria in the chain of events leading to death through the analysis of postmortem tissues (*23*).

Our study hypothesis is that miRNA levels in plasma are differentially expressed among children with severe and uncomplicated malaria because of the parasite sequestration in vital organs of severely ill children. To identify promising biomarkers for SM, we conducted a small RNA next-generation sequencing study to select miRNAs that were differentially expressed by human brain endothelial (HBE) cells exposed to *P. falciparum* iEs selected for cytoadhesion to ePCR, the main host receptor associated with SM (*9*), compared with HBE cells exposed to noncytoadherent iEs and noninfected erythrocytes (niEs). We also compared children who had SM with children who had UM (Figure 1). miRNAs that were differentially expressed in both analyses, together with the *P. falciparum* biomass-associated miRNAs (correlation coefficient >0.50 [*24*]), were quantitatively confirmed in an independent validation cohort set of children with SM and UM using TaqMan quantitative reverse transcription PCR (qRT-PCR).

**Figure 1:**
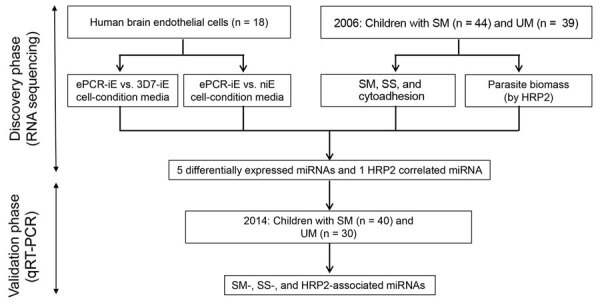
Schematic representation of study design to identify miRNA-based biomarkers of. ePCR, endothelial protein-C receptor (a binding *Plasmodium falciparum* strain-FCR3); HRP2, histidine-rich protein 2; iE, infected erythrocyte; miRNA, microRNA; niE, noninfected erythrocyte; SM, severe malaria; SS, severity symptoms; UM, uncomplicated malaria; 3D7, a nonbinding *P. falciparum* strain.

## Materials and Methods

### Study Population

Plasma samples used to assess miRNA levels were collected in 2 case–control studies conducted in Manhiça District in southern Mozambique during 2006 (n = 113) and 2014 (n = 91). In brief, the cases were children <5 years of age admitted to Manhiça District Hospital for SM and controls were outpatient children with UM (Appendix). The National Mozambican Ethical Review Committee (Mozambique) and Hospital Clínic (Barcelona, Spain) approved study protocols for each of the case–control studies. A signed written informed consent was obtained from each participant’s guardian or parent during the original studies.

### Parasitological Determinations

We prepared thick and thin blood films to quantify *P. falciparum* parasitemia. We used approximately half of a 60μL dried blood drop on Whatman-903 filter paper to extract parasite DNA and performed real-time quantitative PCR (qPCR) targeting the *P. falciparum* 18S rRNA gene (*25**,**26*). HRP2 levels were quantified using commercially available ELISA kits and an in-house highly sensitive quantitative bead suspension array (qSA) based on Luminex technology (Appendix).

### *P. falciparum* Cytoadhesion Assays

We performed cytoadhesion assays to discover the differential expression of miRNAs (Appendix). HBE cells were incubated with *P. falciparum* iEs at the trophozoite stage of the ePCR binding FCR3 strain (ePCR-iE, which expresses the PfEPM1 protein that binds to ePCR receptor) and the 3D7 strain (3D7-iE, a strain without the protein that binds to ePCR receptor). Noninfected erythrocytes were used as negative control. The cell-conditioned media of each group were collected after 1 h (t1) and 24 h of stimulation (t24) and subjected to RNA extraction followed by small-RNA sequencing.

### Molecular Procedures, Gene Target Prediction and Data Analysis

RNA was extracted from cell-conditioned media (3 mL) by using the miRNeasy tissues/cells kit (QIAGEN, https://www.qiagen.com) and from plasma samples (1 mL) by using the miRNeasy plasma/serum kit, with the use of 5µg UltraPure glycogen/sample. Given that the plasma samples were conserved in heparin, RNA was precipitated with lithium chloride as described previously (*27*). Purified RNA was subjected to library preparation, pooling, and sequencing using a HiSeq 2000 (Illumina, https://www.illumina.com) platform, following the protocol for small RNAs (*28*) (Appendix). We used a previously published pipeline (*28*) to assess the sequencing quality, identification, and quantification of small RNAs, normalization and other species RNA contamination (Appendix). To detect miRNAs and isomiRs, reads were mapped to the precursors and annotated to miRNAs or isomiRs using miRBase version 21 with the miraligner (*29*). DESeq2 R package version 1.10.1 (R3.3.2; https://www.r-project.org/about.html) (*30*) was used to perform an internal normalization.

In the 2014 study, we used 50 µL of plasma with no hemolysis for RNA extraction as described, then conducted qRT-PCR (Appendix). We calculated miRNA relative expression levels (RELs) by the 2^−ΔC^_t_ method, where ∆C_t_ = cycle threshold (C_t_) (miRNA) – mean C_t_ (endogenous controls; ECs), considering efficiencies of 100% for all the miRNAs and ECs (*31*).

The selected miRNAs were screened through different gene target prediction programs such as DIANA-microT-CDS (http://www.microrna.gr/microT-CDS), MiRDIP (http://ophid.utoronto.ca/mirDIP), MirGate (http://mirgate.bioinfo.cnio.es), and TargetScan (http://www.targetscan.org) (Appendix). We assessed differential expression of miRNAs and isomiRs using DESEq2 and IsomiRs packages in R (*29**,**32*) (Appendix). All statistical analyses were performed using R version 3.3.2, and graphs were prepared with GraphPad version 6 (https://www.graphpad.com).

## Results

### Discovery Phase

#### miRNA Expression by HBE Cells

**Figure 2:**
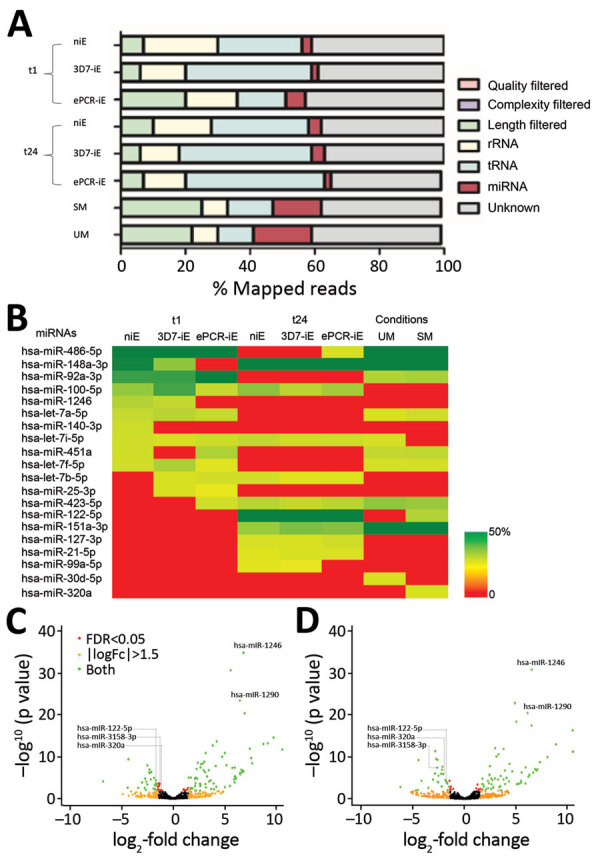
RNA sequencing of human brain endothelial (HBE) cell media and plasma from children recruited in 2006, Mozambique. A) Percentage of mapped reads in different species of small RNAs, for both in vitro and ex vivo approaches. B) Ten most expressed miRNAs in HBE cell medias and plasmas. Color-coded cells show the percentage of each assay/condition (columns) for each miRNA (rows). C) Volcano plot of differentially expressed miRNAs in cell-condition media of niEs versus cell-condition media of iEs with the FCR3-ePCR strain (ePCR-iE) incubated with HBE cells. D) Volcano plot of differentially expressed miRNAs in cell-condition media of iEss with 3D7 strain (3D7-iE) versus cell-condition media of iEs with the FCR3-ePCR strain (ePCR-iE) incubated with HBE cells. Comparisons depicted in C and D were adjusted for multiple testing by the Benjamini-Hochberg method. Negative log_2_-fold change indicates overexpression in ePCR-iE samples. ePCR, endothelial protein-C receptor (a binding *Plasmodium falciparum* strain); HRP2, histidine-rich protein 2; iE, infected erythrocyte; miRNA, microRNA; SM, severe malaria; UM, uncomplicated malaria.

The ePCR binding *P. falciparum* strain (FCR3; ePCR-iE) showed higher levels of cytoadhesion to HBE cells (mean 32.60, SD 4.87 iE/500 cells) than a nonbinding *P. falciparum* (3D7; 3D7-iE) strain (mean 3.20, SD 1.06 iE/500 cells; p = 0.001) and noninfected erythrocytes (mean 3.12, SD 0.39 iE/500 cells; p = 0.001) (Appendix Figure 1). We sequenced 3 replicates of the media collected from each cytoadhesion assay after 1 h (t1) and 24 h (t24), giving a total of >200 million reads/lane, with a mean of 12.10 million reads (SD 13.31) per sample (Table 1; Figure 2, panel A; Appendix Table 1). The mean percentage of miRNAs in the media samples analyzed was 4.01% (SD 2.93%); a mean of 203 (SD 93.82, range 101–465) distinct miRNAs were detected (Appendix Table 1). The 10 most expressed miRNAs for all samples at t1 and t24 time points are described in Figure 2, panel B. No contamination with RNA from other species was observed.

**Table 1:** Quality control and mapped reads in different species of small RNAs from cell-conditioned media of human brain endothelial cells and plasma samples in children with uncomplicated and severe malaria, Mozambique*

Read type		Cell condition								UM, n = 39	SM, n = 44
		niE			3D7-iE			ePCR-iE			
		t1, n = 3	t24, n = 3		t1, n = 3	t24, n = 3		t1, n = 3	t24, n = 3		
Total reads, millions (SD)		8.70 (3.55)	16.71 (14.59)		10.43 (3.48)	25.86 (28.14)		4.78 (2.13)	6.11 (1.18)	10.90 (9.69)	9.26 (6.06)
Quality filtered, counts (SD)		46.00 (36.72)	33.33 (29.67)		14.67 (23.69)	125.67 (217.66)		10.67 (2.31)	16.33 (25.70)	557.62 (1,200.76)	615.75 (1,163.62)
Complexity filtered, counts (SD)		910.67 (775.48)	745.00 (659.60)		369.33 (567.40)	3168.67 (5,438.11)		220.67 (163.57)	308.00 (526.55)	535.97 (884.46)	506.23 (455.16)
Size filtered, millions (SD)		0.63 (0.34)	2.26 (2.99)		0.68 (0.40)	2.12 (2.92)		0.90 (0.48)	0.49 (0.50)	1.94 (1.51)	2.39 (1.82)
Good-quality reads†											
Millions (SD)		8.07 (3.35)	14.44 (11.60)		9.75 (3.10)	23.74 (25.23)		3.88 (2.00)	5.62 (0.84)	8.96 (8.89)	6.88 (4.64)
Percentage (SD)		92.62 (2.54)	90.35 (6.98)		93.93 (2.37)	94.15 (3.26)		79.60 (8.86)	92.62 (6.35)	77.76 (15.31)	74.95 (11.08)
miRNA											
Millions (SD)		0.26 (0.19)	1.09 (1.57)		0.27 (0.19)	0.98 (1.14)		0.25 (0.07)	0.15 (0.13)	2.05 (2.50)	1.33 (1.42)
Percentage (SD)		3.02 (1.73)	4.97 (4.92)		2.47 (1.52)	3.75 (0.54)		7.41 (3.44)	2.47 (1.97)	22.43 (16.01)	20.21 (13.22)
rRNA											
Millions (SD)		2.34 (1.82)	3.12 (2.71)		1.57 (1.72)	5.74 (9.19)		0.72 (0.38)	0.90 (1.08)	0.92 (0.97)	0.81 (0.72)
Percentage (SD)		24.72 (16.01)	20.36 (14.62)		14.84 (15.37)	13.41 (15.42)		19.55 (5.14)	15.13 (16.99)	11.11 (7.75)	11.49 (5.78)
tRNA											
Millions (SD)		1.72 (0.58)	3.37 (1.51)		3.75 (1.80)	6.35 (3.00)		0.84 (0.64)	2.47 (1.47)	1.13 (1.17)	1.14 (0.94)
Percentage (SD)		27.51 (23.37)	32.53 (27.16)		41.04 (20.74)	43.47 (23.59)		18.65 (9.43)	45.24 (26.80)	13.93 (6.85)	17.79 (7.70)
Unknown											
Millions (SD)		3.75 (1.92)	6.86 (6.80)		4.16 (1.67)	10.66 (12.55)		2.07 (0.97)	2.11 (0.67)	4.87 (5.88)	3.59 (2.62)
Percentage (SD)		44.76 (6.35)	42.14 (11.63)		41.65 (5.12)	39.37 (8.36)		54.40 (3.75)	37.15 (7.89)	52.53 (16.01)	50.51 (13.55)

*Reads were obtained from cell-conditioned media of human brain endothelial cells exposed to cytoadherent *P. falciparum–*infected and noninfected erythrocytes, and plasma of Mozambique children with SM and UM. Three replicates of the media were collected from each cytoadhesion assay after 1 (t1) and 24 (t24) hours. ePCR-iE, adherent FCR3 expression endothelial receptor of protein C-infected erythrocytes; miRNA, microRNA; niE, noninfected erythrocytes; SM, severe malaria; UM, uncomplicated malaria; 3D7-iE, nonadherent 3D7-infected erythrocytes. †Reads after filtering low quality, low complexity, and short (<18-nt) sequences.

One hour after incubating the HBE cells with *P. falciparum* infected and noninfected erythrocytes, 111 miRNAs were found to be differentially expressed in cell-condition media of niE and ePCR-iE; 76 of them were downregulated and 35 upregulated in ePCR-iE compared with niE (Figure 2, panel C; Appendix Table 2). At this same time point, 100 miRNAs were differentially expressed in cell-condition media of 3D7-iE and ePCR-iE; 67 were downregulated and 33 upregulated in ePCR-iE compared with 3D7-iE (Figure 2, panel D; Appendix Table 3). Overall, 89 miRNAs were differentially expressed in ePCR-iE compared with both niE and 3D7-iE; 28 of those were upregulated and 61 downregulated in ePCR-iE. There were no differentially expressed miRNAs between niE and 3D7-iE cell-condition media. At t24, only hsa-miR-451a was significantly upregulated in cell-condition media of ePCR-iE with respect to niE (p<0.001) and 3D7-iE (p = 0.023). We found no significantly different miRNAs between niE and 3D7-iE cell-condition media. All differentially expressed isomiRs originated from the selected miRNAs; none of them presented any modifications in the seed region.

#### miRNAs Expression in Plasmas from Children with Malaria of Varying Severity

Out of 113 plasma samples collected from children with SM (N = 57) and UM (N = 56) in Mozambique in 2006, 11 samples were discarded, 5 because of hemolysis (OD_414_>0.2) (*33*) and 6 because no peak was observed between 133–150 nt (typical size for miRNAs plus library adaptors) on the bioanalyzer results after library preparation. Among the 102 sequenced samples (SM = 53, UM = 49), 19 samples (9 SM, 10 UM) were further excluded because of the low number of miRNA reads (<10,000 reads). In total, samples from 83 children (44 with SM and 39 with UM) were included in the analysis (Table 2). 

**Table 2:** Characteristics of children with severe and uncomplicated malaria recruited for case–control studies in 2006 and 2014, Mozambique*

Characteristic	2006				2014		
	UM, n = 39	SM, n = 44	p value		UM, n = 30	SM, n = 40	p value
Age, y, mean (SD)†	2.3 (1.1)	2.4 (1.3)	0.671		2.2 (1.3)	2.8 (1.2)	0.419
Sex, no. (%)							
M	24 (62)	28 (64)	1.000		18 (60)	21 (52.5)	0.532
F	15 (38)	16 (36)			12 (40)	19 (47.5)	
HRP2, ng/mL, GM (SD)	71.3 (10.7)	331.4 (40.7)	<0.001		24.1 (4.9)	78.7 (12.2)	0.038
qPCR, parasites/μL, GM (SD)	2,084.9 (302.5)	7,976.1 (1,079.6)	0.004		72,845.9 (7,193.9)	94,099.6 (8,716.0)	0.549
Splenomegaly, no. (%)							
No	33 (85)	21 (48)	0.001		ND	27 (67.5)	NA
Yes	6 (15)	23 (52)			ND	13 (32.5)	
Hepatomegaly, no. (%)							
No	38 (97)	35 (80)	0.016		ND	35 (87.5)	NA
Yes	1 (3)	9 (20)			ND	5 (12.5)	
Hyperlactatemia, no. (%)							
No	10 (26)	5 (11)	0.152		26 (86.7)	27 (67.5)	0.064
Yes	29 (74)	39 (89)			4 (13.3)	13 (32.5)	
Temperature, ºC, mean (SD)	38.0 (1.6)	38.5 (1.1)	0.093		38.0 (1.3)	38.2 (1.4)	0.437
Weight, kg, mean (SD)	11.3 (2.8)	11.0 (2.8)	0.599		12.3 (2.9)	12.7 (3.3)	0.476
Platelets, 10^9^/L, mean (SD)	156.7 (86.8)	115.8 (66.8)	0.018		149.0 (89.7)	95.3 (69.3)	0.001
Glucose, mM, mean (SD)‡	6.2 (1.5)	5.9 (1.8)	0.391		6.6 (1.3)	6.0 (2.6)	0.165
WBC, 10^9^/L, mean (SD)	9.9 (4.1)	10.2 (3.9)	0.774		9.7 (3.8)	9.6 (5.0)	0.929
Neutrophils, %, mean (SD)§	54.1 (16.7)	54.4 (14.3)	0.940		50.7 (20.6)	58.9 (13.7)	0.447
Lymphocytes, %, mean (SD)¶	39.4 (17.9)	36.3 (12.6)	0.374		26.1 (17.1)	25.6 (12.2)	0.995
Lactate, mM, mean (SD)	3.0 (1.7)	4.7 (3.6)	0.009		2.8 (2.2)	3.6 (2.4)	0.035
Severe malaria syndromes, no. (%)							
Prostration		33 (75.0)				30 (75.0)	
Acute respiratory distress		18 (40.9)				19 (47.5)	
Severe anemia		17 (38.6)				7 (17.5)	
Multiple seizures		11 (25.0)				24 (60.0)	
Cerebral malaria		2 (4.5)				7 (17.5)	
Hypoglycemia		2 (4.5)				2 (5.0)	

*Data were gathered in a discovery study in 2006 and validation study in 2014. GM, geometric mean; HRP2, histidine-rich protein 2: NA, not applicable; ND, not determined; SM, severe malaria; UM, uncomplicated malaria; WBC, white blood cells. †No data for 1 sample (UM = 1) in 2014 study. ‡No data for 3 samples (SM = 2; UM = 1) in 2014 study. §No data for 4 samples (SM = 4) in 2014 study. ¶No data for 3 samples (SM = 3) in 2014 study.

The sequencing of the 83 plasma samples yielded a mean of 9.42 (SD 6.4) million reads per sample (Table 1; Figure 2, panel A; Appendix Table 4). The mean percentage of miRNAs per plasma samples was 20.5% (SD 13.2%), with a mean of 395 (SD 169, range 116–786) distinct miRNAs detected (Appendix Table 4). The total number of miRNAs detected across samples was 1,450. The 10 most expressed miRNAs can be found in Figure 2, panel B. No contamination with RNA from other species was observed.

**Figure 3:**
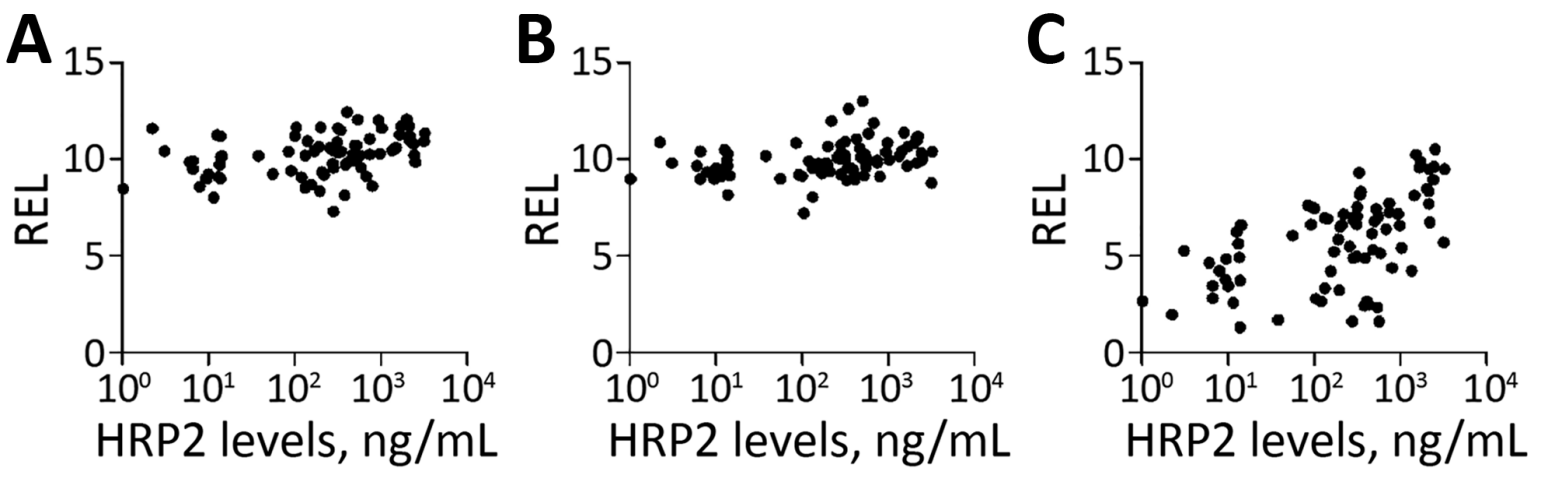
Spearman correlations between HRP2 levels and relative expression levels (RELs) of 3 miRNA in plasma samples from children with malaria, 2006, Mozambique. A) hsa-miR-10b-5p; B) hsa-miR-378a-3p; C) hsa-miR-4497. HRP2 levels and miRNA RELs were log transformed. The correlation analysis was adjusted for multiple testing by the Benjamini-Hochberg method. HRP2, histidine-rich protein 2; miRNA, microRNA; REL, relative expression levels.

We found hsa-miR-122–5p upregulated in children with SM (Table 3). In the subanalysis by signs of severity, 5 miRNAs were associated with severe anemia (SA), prostration, and acute respiratory distress (ARD) (Table 3). Twelve miRNAs were associated with PM-agglutination and cytoadhesion to g1CqR (Table 3). We observed no associations between miRNA counts and other cytoadhesion data such as rosetting and binding to CD36 and to CD54. After adjusting for multiple comparisons, we found 3/1,450 miRNAs identified in RNA sequencing data, hsa-miR-10b-5p, hsa-miR-378a-3p, and hsa-miR-4497, correlated with HRP2 levels determined by qSA Spearman analysis (Figure 3). We observed similar correlations when HRP2 levels were determined by ELISA (Appendix Table 5). miRNAs were neither associated with hepatomegaly nor with splenomegaly. All differentially expressed isomiRs between children with SM and those with UM belong to the differentially expressed miRNAs, with no modifications in the seed region.

**Table 3:** Association of miRNA levels with severe malaria, symptoms of severity, and *Plasmodium falciparum* cytoadhesion among children with uncomplicated and severe malaria, Mozambique*

Characteristic	miRNA	baseMean	log_2_-fold change	Adjusted p value
Clinical data				
SM, n = 44 vs. 39 UM				
All	hsa-miR-122-5p	19,929.69	1.67	0.001
SA, n = 17 vs. 39 UM				
	hsa-miR-4492	17.34	2.81	0.046
	hsa-miR-4497	293.66	2.18	0.046
Prostration, n = 33 vs. 39 UM				
	hsa-miR-122-5p	20,677	1.89	0.001
	hsa-miR-6087	5.36	2.39	0.033
	hsa-miR-511-5p	126.67	1.36	0.040
Acidosis or respiratory distress, n = 18 vs. 39 UM				
	hsa-miR-122-5p	13,367.43	2.21	<0.001
	hsa-miR-4497	272.39	2.05	0.07
Cytoadhesion data				
Platelet-mediated agglutination, n = 50 vs. 19 UM				
	hsa-miR-3158-3p	1,180.96	−2.26	<0.001
	hsa-miR-320a	22,005.69	−1.48	0.001
	hsa-miR-4492	18.33	2.78	0.002
	hsa-miR-1290	1,011.34	−1.38	0.014
	hsa-miR-320b	1,191.44	−1.23	0.014
	hsa-miR-320c	408.32	−1.29	0.014
	hsa-miR-1246	3,907.45	−1.32	0.019
	hsa-miR-6741-5p	48.11	−1.81	0.023
	hsa-miR-1228-5p	82.73	−1.88	0.023
	hsa-miR-3195	16.35	2.21	0.023
	hsa-miR-7706	334.86	−1.00	0.023
gC1qR, n = 35 vs. 34 UM				
	hsa-miR-1-3p	622.35	2.09	0.003

*Positive fold change indicates overexpression in severe malaria and symptoms of severity compared to UM as well as parasites showing cytoadhesion compared to none. Total number of miRNAs in RNA sequencing data was 1,450. p value adjusted for multiple testing by the Benjamini-Hochberg method. baseMean, mean normalized expression of the miRNAs in all the samples; miRNA, microRNA; SA, severe anemia, SM, severe malaria; UM, uncomplicated malaria.

### Validation Cohort

Among the 89 miRNAs differentially expressed in cell-condition media of HBE cells exposed to niE and 3D7-iE compared with ePCR-iE, we confirmed 5 miRNAs to be differentially expressed between children with SM and UM. These 5 miRNAs (hsa-miR-122–5p, hsa-miR-320a, hsa-miR-1246, hsa-miR-1290 and hsa-miR-3158–3p), along with hsa-miR-4497 miRNA, which had a correlation coefficient with HRP2 >0.5 (Figure 3), were selected for TaqMan qRT-PCR validation in an independent cohort of children with SM and UM recruited in 2014. Among the 91 plasma samples collected from these children, 21 were discarded because of hemolysis (OD_414_>0.2) (*33*). Of the 70 remaining samples, 40 were collected from children with SM and 30 from children with UM (Table 2). 

All samples tested by qRT-PCR amplified the exogenous control (ath-miR-159a) with a C_t_ value<18 and a coefficient of variance (CV) <5%, suggesting the correct RNA extraction and cDNA preparation. We selected 3 ECs, hsa-miR-191–5p (CV = 4.8%, baseMean = 3953.3, log_2_-fold change [FC] −0.02, SD 0.56), hsa-miR-30d-5p (CV = 4.9%, baseMean = 14172.31, FC 0.01, SD 0.61), and hsa-miR-148a-3p (CV = 5%, baseMean = 111593.08, FC 0.11, SD 0.82) as a panel for qRT-PCR analysis. Among these, the NormFinder stability value was 0.044 for the combination of hsa-miR-30d-5p and hsa-miR-191–5p, and thus we selected those 2 ECs. No statistically significant differences were found when we compared C_t_ values of the exogenous controls and 2 endogenous controls between SM and UM samples (Appendix Figure 2). We performed standard curves for all miRNAs (ECs and selected miRNAs), giving efficiencies of 91.1%–103.8% (Appendix Table 6), which were assumed as 100% to calculate the relative expression values using the 2^−ΔC^_t_ method (*31*).

**Figure 4:**
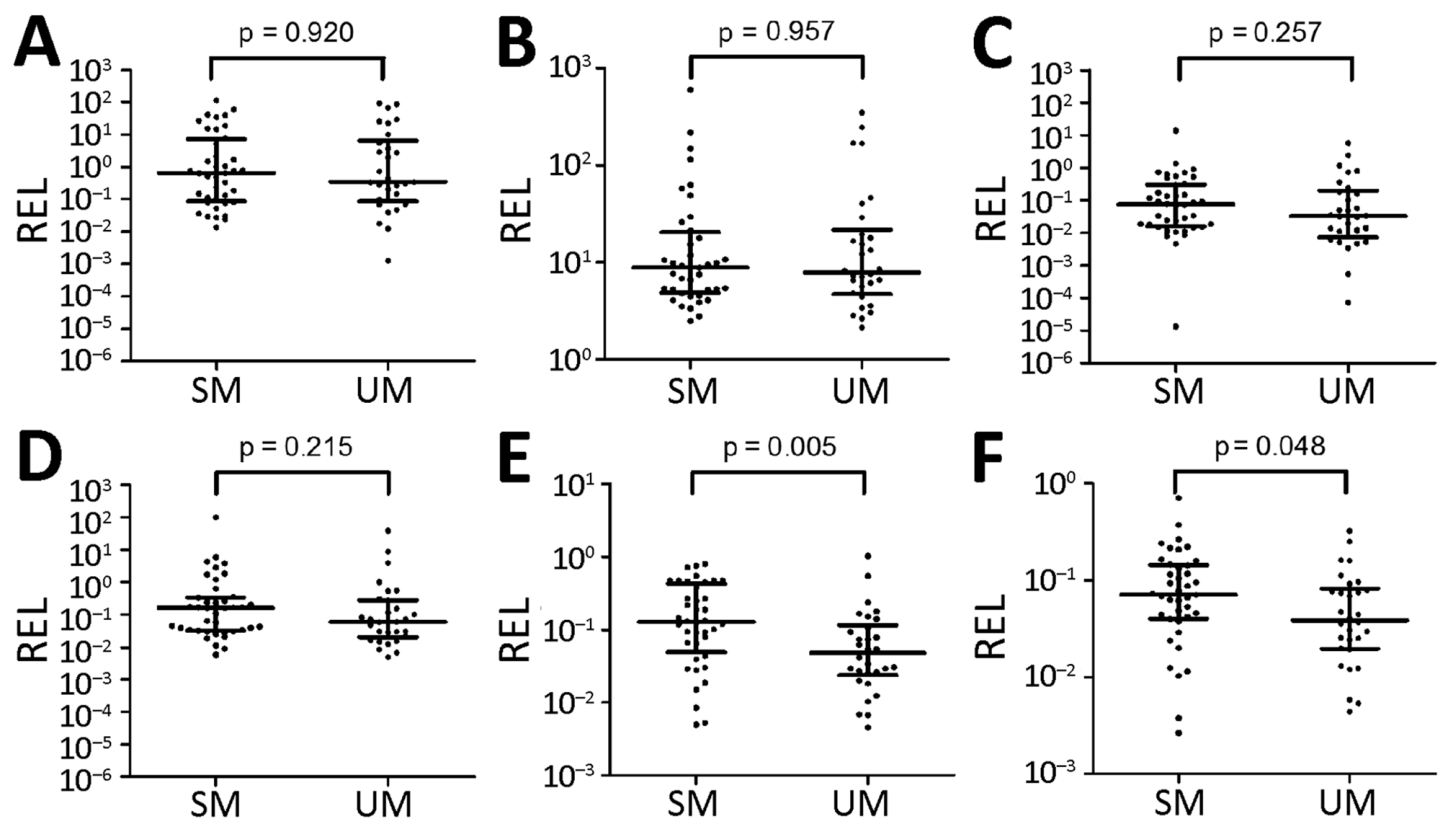
MiRNA validation in plasma samples of children with malaria, 2014, Mozambique. A) hsa-miR-122-5p; B) hsa-miR-320a; C) hsa-miR-1246; D) hsa-miR-1290; E) hsa-miR-3158-3p; F) hsa-miR-4497. RELs were calculated with respect to the mean of 2 endogenous controls (hsa-miR-30d-5p and hsa-miR-191–5p) and compared between children with SM and UM. Statistical differences were obtained by using the Mann-Whitney U test. Error bars represent medians and interquartile ranges. HRP2, histidine-rich protein 2; miRNA, microRNA; REL, relative expression levels; SM, severe malaria; UM, uncomplicated malaria.

**Figure 5:**
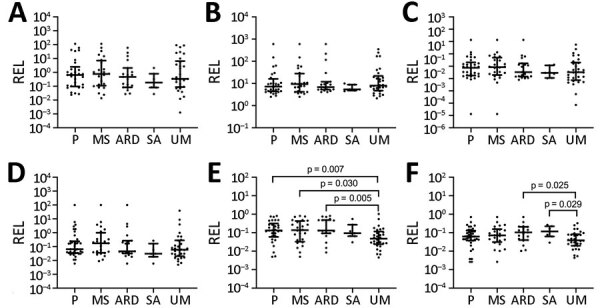
Association of microRNA levels with symptoms of severity in children with malaria, Mozambique, 2014. A) hsa-miR-122-5p; B) hsa-miR-320a; C) hsa-miR-1246; D) hsa-miR-1290; E) hsa-miR-3158-3p; F) hsa-miR-4497. RELs were calculated with respect to the mean of 2 endogenous controls (hsa-miR-30d-5p and hsa-miR-191–5p) and compared between children with UM and symptoms of severity. Distributions were compared using Mann-Whitney U test. Error bars represent medians and interquartile ranges. p values are shown for significant comparisons. ARD, acidosis or acute respiratory distress; MS, multiple seizures; P, prostration; REL, relative expression levels; SA, severe anemia; UM, uncomplicated malaria.

**Figure 6:**
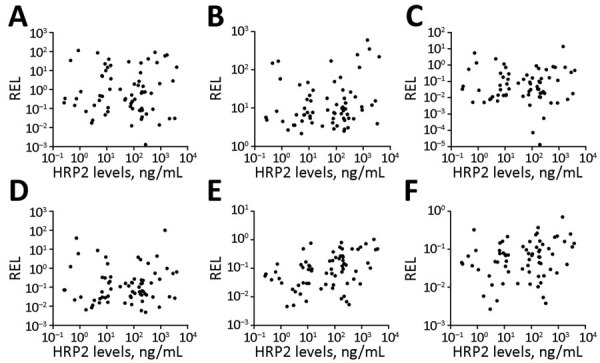
Spearman correlations between HRP2 levels and microRNA RELs in plasma samples from children with malaria, Mozambique, 2014. A) hsa-miR-122-5p; B) hsa-miR-320a; C) hsa-miR-1246; D) hsa-miR-1290; E) hsa-miR-3158-3p; F) hsa-miR-4497. HRP2 levels and microRNA RELs were log transformed. HRP2, histidine-rich protein 2; REL, relative expression levels.

The relative expression levels of hsa-miR-3158–3p and hsa-miR-4497 were significantly higher in children with SM than UM (p<0.05) (Figure 4). We found that hsa-miR-3158–3p levels were higher in children who had prostration, multiple seizures, and ARD compared with those who had UM (p<0.05; Figure 5). Severe anemia and ARD symptoms were associated with higher hsa-miR-4497 levels (p<0.05; Figure 5). No such associations were observed for cerebral malaria and hypoglycemia. RELs of hsa-miR-3158–3p and hsa-miR-4497 were found positively correlated with HRP2 levels quantified by qSA (p<0.05; Figure 6). Similar correlations were observed when HRP2 levels were determined by ELISA (Appendix Table 5).

### miRNA Gene Target Prediction

We identified a total of 87 putative targets for hsa-miR-3158–3p and hsa-miR-4497 miRNAs, none of which were shared by both miRNAs (Appendix Table 7). We predicted 45 experimentally validated mRNA targets for hsa-miR-3158–3p and 42 for hsa-miR-4497; the predicted targets were found to be involved in a broad range of biologic processes (Appendix Table 8). However, significance was lost when adjusted by the Benjamini-Hochberg method; none of the target genes were clustered under the KEGG pathway with p<0.05.

## Discussion

Because of their specificity to cell type (*17*), microRNAs can reflect disease states and organ damage. Consequently, they have the potential to provide a new screening method for early detection of pathological *P. falciparum* sequestration and could become an effective prognosis tool for severe malaria. Moreover, the detection of miRNAs associated with organ damage in host biofluids may provide an alternative to postmortem autopsies for determining the presence of parasites in host vital organs. This approach creates new opportunities to develop malaria diagnostic tools that can guide treatment decisions, and to understand the role of human miRNAs in several disease conditions (*23*).

In the discovery phase, 89 miRNAs were found to be differentially expressed in the media of HBE cells after incubation with an ePCR-cytoadherent *P. falciparum* strain compared with noncytoadherent parasites and noninfected erythrocytes. In addition, 15 miRNAs in plasma samples obtained from children were associated with SM, with specific severity symptoms, and with the cytoadherent *P. falciparum* phenotype, compared with UM and noncytoadherent parasites. In the validation phase, we confirmed the higher abundance of hsa-miR-3158–3p and hsa-miR-4497 in children with SM than in children with UM. Prostration, multiple seizures, SA, and ARD symptoms of severity were associated with higher levels of hsa-miR-3158–3p and hsa-miR-4497. hsa-miR-4497 levels were also positively correlated with the parasite biomass as quantified by the levels of HRP2 in both the discovery and validation phases. Overall, these findings suggest that different physiopathological processes in SM and UM lead to differential expression of miRNAs in plasma.

HBE cells released a high number of the miRNAs when they were stimulated with an ePCR binding *P. falciparum* strain within the first hour of incubation. After 24 hours, the system stabilized; 1 miRNA (hsa-miR-451a) was found at higher levels in cell-conditioned media of HBE cells incubated with an ePCR binding strain than in cells stimulated with nonadherent (3D7-iE) or noninfected erythrocytes. miR-451 has been implicated in translocation to form a chimera with *Plasmodium* mRNAs to block their translation (*34*) and was also found to be abundant in sickle erythrocytes (*35*). In addition, it has been shown that parasites could reduce miR-451 levels in host fluids (*36*). However, this finding was not confirmed in plasmas from the children in this study. Five miRNA levels were higher in children with SM and severity symptoms (prostration, SA, and ARD) than in children with UM. *P. falciparum* cytoadhesion phenotypes (PM-agglutination and cytoadhesion to gC1qR) were also associated with the differential expression of miRNAs, suggesting that the interaction between PfEMP1 and host receptors leads to the secretion to plasma of specific miRNAs. Moreover, 3 miRNAs (hsa-miR-10b-5p, hsa-miR-378a-3p, and hsa-miR-4497) were positively correlated with HRP2 levels.

We selected 6 candidate miRNAs identified in the discovery phase to determine the validity of the previous results in an independent cohort of children in Mozambique. The relative expression of hsa-miR-3158–3p and hsa-miR-4497 was significantly higher in children with SM than in those with UM; hsa-miR-3158–3p levels were higher in children with prostration, multiple seizures, and ARD, and hsa-miR-4497 in children with SA and ARD. To our knowledge, hsa-miR-3158–3p, which is widely expressed in skin, spleen, kidney, and brain tissues (*37*), has been associated with bipolar disorders (*38*) but not with other infectious diseases. Further validation is required for hsa-miR-3158–3p because the levels of this miRNA were found to be downregulated in the plasma from children recruited in 2006 with positive PM-agglutination compared with no PM-agglutination, a *P. falciparum* cytoadhesion phenotype which has been associated with malaria severity (*39*). However, the positive correlation of hsa-miR-4497 with HRP2 levels, which was consistently observed in the cohorts of children from 2006 and 2014, suggested that increasing parasite biomass associated with parasite sequestration may lead to higher levels of secretion of this specific miRNA by damaged tissues. The miRNA hsa-miR-4497 is widely expressed in the lymph nodes and spleen, kidney, and liver tissues (*37*). Overall, this study shows that hsa-miR-4497, which is also associated with SM, might be an interesting proxy marker of malaria severity. However, hsa-miR-4497 has been identified as a tumor suppressor (*40*) and associated with *Mycobacterium tuberculosis* infection (*41*). Therefore, longitudinal studies are required to assess the prognostic value of this miRNA, as well as to estimate its differential expression in children with severity due to nonmalarial infections.

Few of the most expressed miRNAs found in our study, which represent 70% of the total miRNA counts in plasma samples, have been previously reported as highly abundant in plasma samples (*28**,**42*). According to public data deposited in the miRmine database (*43*), hsa-miR-486–5p and hsa-miR-451a are the 2 most abundant miRNAs in plasma; both were among the 10 most expressed miRNAs in our study. Although no data are available on miRNAs from cell-conditioned media of HBE cells, miRNA data from other cell types, such as primary tissue explants, primary stromal cells, and breast cancer cell lines, also show low miRNA yield (*44*), similar to this study. Our observation indicates that RNA sequencing data obtained in this study is of good quality and can be used for posterior analysis with high confidence. 

The first limitation of our study is that we used only HBE cells and ePCR binding parasites for the in vitro assay and therefore may have missed miRNAs produced by other parasite-host interactions contributing to SM. Second, plasma samples used in this study were collected retrospectively. Therefore, factors before small RNA sequencing and TaqMan- qRT-PCR, such as time taken between centrifugation, storage, and storage temperature, might have varied among the samples, affecting miRNA plasma levels (*45**,**46*). However, confirmation of findings in both the study cohorts suggest a minimal effect of preanalysis conditions in the results. Third, variations in the number of miRNAs identified in replicates of in vitro experiments may have led to the loss of some miRNAs. Fourth, the lack of tissue samples from organs with *P. falciparum* sequestration restricted the histological confirmation of identified miRNAs, and the presence of co-infections other than blood culture positive bacteremia cannot be neglected in the studied plasma samples. Finally, the association of each miRNA with specific symptoms that are part of the SM case definition may need further validation using a larger sample size, considering that our numbers were relatively small for individual SM criteria. In addition, future studies using machine-learning approaches would enable the identification of a combination of miRNAs that may detect SM pathologies.

In conclusion, the profiling of miRNAs in media from HBE cells after incubation with a cytoadherent *P. falciparum* strain and in plasma from children with different clinical manifestations enabled us to identify promising miRNA candidates for characterizing severe malaria, specifically hsa-miR-4497. This study is a base for future analyses to understand the value of these miRNAs as a prognostic biomarker and for disentangling the etiology of SM.

Appendix Additional information about plasma microRNA profiling of *Plasmodium falciparum* biomass and association with severity of malaria disease in children in Mozambique.
