# Supplementary material for: Plasma MicroRNA Profiling of Plasmodium falciparum Biomass and Association with Severity of Malaria Disease
Source: Emerg Infect Dis. 2021 Feb;27(2):430–42. doi: 10.3201/eid2702.191795 (PMC7853565; doi:10.3201/eid2702.191795)
Supplement: Appendix — Additional information about plasma microRNA profiling of Plasmodium falciparum biomass and association with severity of malaria disease in children in Mozambique. [file 19-1795-Techapp-s1.pdf]

Article DOI: https://doi.org/10.3201/eid2702.191795

Plasma MicroRNA Profiling of *Plasmodium falciparum* Biomass and Association with Severity of Malaria Disease

Appendix

Material and Methods

Study Population

Clinical malaria was defined as the presence of fever (axillary temperature ≥37.5°C) with an asexual parasitemia of *Pf* ≥500/µL. Children with severe malaria (SM) were those with ≥1 of the following symptoms: cerebral malaria (Blantyre Coma Score ≤2), severe anemia (SA; packed cell volume <15% or hemoglobin <5 g/dL), acidosis or acute respiratory distress (ARD; lactate >5 mM and/or chest indrawing or deep breathing), prostration (inability to sit or breastfeed in children old enough to do so based on their age), hypoglycemia (blood glucose <2.2 mM), and multiple seizures (≥2 convulsions in the preceding 24 h) following the modified World Health Organization criteria (*1*). Children with uncomplicated malaria (UM) were those with clinical malaria but not manifesting any signs or symptoms of severity mentioned above (*2*). The presence of concomitant bacteremia was tested in all SM cases using blood cultures; and children with positive bacteremia were excluded. Children with SM were treated according to Mozambican national guidelines with parenteral quinine in 2006 or parenteral artesunate (complemented with an oral artemisinin-based combination therapy) in 2014, and those with UM were treated with a combination of oral amodiaquine and sulfadoxine−pyrimethamine (Fansidar; Roche Pharma (Schweiz) AG, https://www.roche.ch/pharma.htm) in 2006 or with artemether-lumefantrine (Coartem; Novartis, https://www.novartis.com) in 2014. We collected 10 mL of heparinized blood from study participants and processed within 2 hours after collection. Filter paper dried blood spots of 60µL blood were prepared from the vacutainer blood. After centrifugation at 1,000 rpm for 10 min at 4°C, plasma was stored at −20°C. The 2014 study was conducted as a quasi-exact repetition of the 2006 study; the only difference was that cases and

controls were matched by parasitemia level. We assessed biochemistry parameters (glucose and lactate) using Vitros DT60-II (Ortho Clinical Diagnostics, https://www.orthoclinicaldiagnostics.com and a full blood count using Sysmex Kx21 analyzers (Sysmex, https://www.sysmex.co.jp) for each patient.

Parasitological Determinations

Histidine-rich protein 2 (HRP2) levels were quantified using commercially available enzyme-linked immunosorbent assay kits (Malaria Ag CELISA; Cellabs Pty. Ltd, https://www.cellabs.com.au) and an in-house highly sensitive quantitative bead suspension array based on Luminex technology. In brief, plasma samples were incubated overnight at 4°C with 2,000 magnetic beads to a final dilution of 1:10. After washing, beads were sequentially incubated with 100 µL of in-house biotinylated antibody α-HRP2 (MBS832975, MyBiSource, https://www.mybiosource.com) at 1 µg/mL and with streptavidin-PE (42250–1ML, Sigma Aldrich, https://www.sigmaaldrich.com) at 1:1000 dilution. Finally, beads were washed and resuspended in assay buffer, and the plate was read using the Luminex xMAP 100/200 analyzer (Luminex Corp., https://www.luminexcorp.com). A minimum of 50 microspheres/analyte were acquired and results were exported as crude median fluorescent intensity (MFI). Background (blank) MFIs were subtracted and normalized to account for plate-to-plate variation. Quantification was performed against a 5-parameter logistic regression curve fitted from a calibration curve consisting of recombinant protein HRP2 type A (890015, Microcoat GmbH, https://www.microcoat.de).

Pf Cytoadhesion Assays

Human brain microvascular endothelial cells (reference no. P10361 Innoprot, https://innoprot.com) were cultured in 12-well plates following the supplier's recommendations and were left until 40% confluency was achieved. HBE cells were incubated in triplicate with *Pf*-iEs at trophozoite stage of the ePCR binding FCR3 strain (ePCR-iE, which expresses the PfEPM1 protein that binds to ePCR receptor) and 3D7 strain (3D7-iE, a strain without the protein that binds to ePCR receptor) with 5% of both parasitemia and hematocrit. Noninfected erythrocytes were used as negative control. The cell-conditioned media of each group were collected after 1 h (t1) and 24 h of stimulation (t24). Next day, HBE cells were stimulated as described above, after 1-hour incubation in agitation, cells were washed ≥10 times with binding media and fixed with 2% glutaraldehyde (Sigma) in PBS (Gibco, Thermo Fisher) overnight to

assess adhesion by light microscopy. After washing with water, cells were stained with 10% Giemsa. iE and niE bound were counted in 6 different wells/assay in ≥500 nuclei cell/well. Results were presented as the number of adhered iE per 500 nuclei of cells. Estimation of *Pf* adhesion to purified receptors (CD36, CD54, and g1CqR) as well as platelet-mediated (PM)-agglutination and rosetting was performed as described elsewhere (*2,3*). Cytoadherence was defined as positive only if the number of iEs bound per mm^2 > the mean binding +2 SD to Duffy-Fc coated petri dishes. *Pf* isolates were considered positive for PM clumping if the frequency of clumps was higher in the presence of platelets than in buffer-control and for rosetting if the frequency of rosettes was >2% (*2,4*).

Small RNA Sequencing

Before RNA extraction, the level of hemolysis in plasma samples was assessed by spectrophotometry (EPOCH, BioTek) at a wavelength of 414nm (absorbance peak of free hemoglobin). Samples were classified as nonhemolysed if the optical density at 414nm <0.2 (*5*). RNA was extracted from cell-conditioned media (3 mL) using the miRNeasy tissues/cells kit and plasma samples (1 mL) using miRNeasy plasma/serum kit (both QIAGEN, https://www.qiagen.com), with the use of 5µg UltraPure glycogen/sample (Invitrogen, Thermo Fisher). Given that the plasma samples were conserved in heparin, RNA was precipitated with lithium chloride (LiCl) as described elsewhere (*6*). Purified RNA quality and quantity were determined using the Bioanalyzer (Agilent Technologies, https://www.agilent.com) followed by preparation of libraries using NEBNext Small RNA Library Prep Set for Illumina (New England Biolabs, https://www.neb.com), then separation of libraries in polyacrylamide gels (Novex; Invitrogen). The Bioanalyzer was again used to quantify and assess the size of the libraries. Further, libraries were pooled at the same equimolar concentrations and no more than 18 libraries were sequenced in the same lane using a HiSeq 2000 (Illumina) platform following the protocol for small RNAs (*7*).

A previously published pipeline was used to assess the sequencing quality, identification, and quantification of small RNAs and normalization (*7*). First, a quality control (QC) was conducted using FASTX-Toolkit and FastQ Screen. After adaptor removing, reads with the following features were removed: reads <18 nt; mean PHRED scores <30; and low complexity reads based on the mean score of the read. Good quality reads were then annotated to main RNA categories (tRNA, rRNA, and miRNAs), and miRNA complexity was estimated as the number of

distinct miRNAs observed in each sample. Finally, contamination with RNA from other species was evaluated by mapping reads to clade-specific mature miRNA sequences extracted from miRBase v21 (*8*). The tested species categories include animal sponges, nematodes, insects, lophotrochozoan, echinoderms, fish, birds, reptiles, rodents, and primates.

Sequences that passed the QC were subjected to the seqBuster/seqCluster tool that retrieves miRNA and isomiRs counts (*9,10*). To detect miRNAs and isomiRs, reads were mapped to the precursors and annotated to miRNAs or isomiRs using miRBase version 21 with the miraligner (*9*). DESeq2 R package v.1.10.1 (R version 3.3.2) (*11*) was used to perform an internal normalization in which the counts for a miRNA in each sample were divided by the median of the ratios of observed counts to the geometric mean of each corresponding miRNAs over all samples.

Reverse Transcription Quantitative PCR

We used 50 µL of plasma with no hemolysis from the children recruited in 2014 for RNA extraction as described above. A synthetic RNA mimicking ath-miR-159a (*Arabidopsis thaliana*; Metabion, http://www.metabion.com) was added after lysis reaction at a final concentration of 1.5 pM. cDNA synthesis and RT-qPCR (ABI PRISM 7500 HT Real-Time System; Applied Biosystems) were performed using the TaqMan Advanced miRNA assays. A standard curve of 5 serially diluted points was prepared with cDNA of 6 randomly selected samples and run in triplicate for each miRNA. Results were normalized using a combination of endogenous controls (ECs). The selection of ECs was based on the following criteria: a) reported in scientific literature as previously used as ECs (*12,13*), b) coefficient of variance (CV) of normalized counts across all samples $\leq$5%, c) basemean ≥3000, d) SD $\leq$1, and e) $\log_2$fold change between SM and UM patients $\leq$1. Finally, the best 2 ECs tested as housekeepings using the NormFinder (*14*) were used for normalization of RT-qPCR data. miRNA relative expression levels (RELs) were calculated with the $2^{-\Delta Ct}$ method, where ΔCt = [Ct (miRNA) – Mean Ct (ECs)], considering efficiencies of 100% for all the miRNAs and ECs (*12*).

In silico Analysis

The selected miRNAs were screened through 4 different gene target prediction programs: DIANA-microT-CDS (*15*), MiRDIP (*16*), MirGate (*17*), and TargetScan (http://www.targetscan.org/vert_71). Identified gene targets of each program were compared

using an online tool, Venny2.1.0 (http://bioinfogp.cnb.csic.es/tools/venny). The gene targets that occurred in more than one database were selected and screened through the miRTarBase (*18*) online program to check if these genes have been experimentally validated previously. These gene targets were anticipated to be true positive targets present at detectable levels in field samples. The identified gene targets were further analyzed by DAVID 6.8 using *Homo sapiens* as the reference species. Genes were clustered to Gene Ontology terms and KEGG pathways (fold enrichment >1.5, $p<0.05$).

Statistical Analysis

Differential expression of miRNAs and isomiRs was assessed using DESEq2 and IsomiRs packages in R (*9,10*), which use negative binomial generalized linear models adjusted for multiple testing with the false discovery rate (FDR) by the Benjamini-Hochberg method (*19*). Those with an FDR ≤5% were selected for posterior analysis. Analysis of the modification in the bases of the seed region was carried using isomiR package to determine a possible change in the target messenger RNAs. We performed Mann-Whitney U test to compare continuous data and χ^2 tests to compare categorical data. Spearman correlation analysis was performed to assess the correlation of miRNA RELs (log transformed) with log transformed HRP2 levels. A two-sided $p < 0.05$ was considered statistically significant. All statistical analyses were performed using R 3.3.2 in Linux-based system and graphs were prepared with GraphPad.

References

1. World Health Organization. Guidelines for the treatment of malaria. 3rd ed. 2015 [cited 2020 Aug 6]. https://www.who.int/malaria/publications/atoz/9789241549127

2. Mayor A, Hafiz A, Bassat Q, Rovira-Vallbona E, Sanz S, Machevo S, et al. Association of severe malaria outcomes with platelet-mediated clumping and adhesion to a novel host receptor. PLoS One. 2011;6:e19422. PubMed https://doi.org/10.1371/journal.pone.0019422

3. Roberts DJ, Craig AG, Berendt AR, Pinches R, Nash G, Marsh K, et al. Rapid switching to multiple antigenic and adhesive phenotypes in malaria. Nature. 1992;357:689–92. PubMed https://doi.org/10.1038/357689a0

4. Magallón-Tejada A, Machevo S, Cisteró P, Lavstsen T, Aide P, Rubio M, et al. Cytoadhesion to gC1qR through *Plasmodium falciparum* erythrocyte membrane protein 1 in severe malaria. PLoS Pathog. 2016;12:e1006011. PubMed https://doi.org/10.1371/journal.ppat.1006011

5. Kirschner MB, Edelman JJ, Kao SC, Vallely MP, van Zandwijk N, Reid G. The impact of hemolysis on cell-free microRNA biomarkers. Front Genet. 2013;4:94. PubMed https://doi.org/10.3389/fgene.2013.00094

6. Wang J, Chen J, Chang P, LeBlanc A, Li D, Abbruzzesse JL, et al. MicroRNAs in plasma of pancreatic ductal adenocarcinoma patients as novel blood-based biomarkers of disease. Cancer Prev Res (Phila). 2009;2:807–13. PubMed https://doi.org/10.1158/1940-6207.CAPR-09-0094

7. Rubio M, Bustamante M, Hernandez-Ferrer C, Fernandez-Orth D, Pantano L, Sarria Y, et al. Circulating miRNAs, isomiRs and small RNA clusters in human plasma and breast milk. PLoS One. 2018;13:e0193527. PubMed https://doi.org/10.1371/journal.pone.0193527

8. Griffiths-Jones S, Grocock RJ, van Dongen S, Bateman A, Enright AJ. miRBase: microRNA sequences, targets and gene nomenclature. Nucleic Acids Res. 2006;34:D140–4. PubMed https://doi.org/10.1093/nar/gkj112

9. Pantano L, Estivill X, Martí E. SeqBuster, a bioinformatic tool for the processing and analysis of small RNAs datasets, reveals ubiquitous miRNA modifications in human embryonic cells. Nucleic Acids Res. 2010;38:e34. PubMed https://doi.org/10.1093/nar/gkp1127

10. Pantano L, Estivill X, Martí E. A non-biased framework for the annotation and classification of the non-miRNA small RNA transcriptome. Bioinformatics. 2011;27:3202–3. PubMed https://doi.org/10.1093/bioinformatics/btr527

11. Love MI, Huber W, Anders S. Moderated estimation of fold change and dispersion for RNA-seq data with DESeq2. Genome Biol. 2014;15:550. PubMed https://doi.org/10.1186/s13059-014-0550-8

12. Marabita F, de Candia P, Torri A, Tegnér J, Abrignani S, Rossi RL. Normalization of circulating microRNA expression data obtained by quantitative real-time RT-PCR. Brief Bioinform. 2016;17:204–12. PubMed https://doi.org/10.1093/bib/bbv056

13. Yeri A, Courtright A, Reiman R, Carlson E, Beecroft T, Janss A, et al. Total extracellular small RNA profiles from plasma, saliva, and urine of healthy subjects. Sci Rep. 2017;7:44061. PubMed https://doi.org/10.1038/srep44061

14. Andersen CL, Jensen JL, Ørntoft TF. Normalization of real-time quantitative reverse transcription-PCR data: a model-based variance estimation approach to identify genes suited for normalization, applied to bladder and colon cancer data sets. Cancer Res. 2004;64:5245–50. PubMed https://doi.org/10.1158/0008-5472.CAN-04-0496

15. Paraskevopoulou MD, Georgakilas G, Kostoulas N, Vlachos IS, Vergoulis T, Reczko M, et al. DIANA-microT web server v5.0: service integration into miRNA functional analysis workflows. Nucleic Acids Research. 2013;41:W169–73.

16. Tokar T, Pastrello C, Rossos AEM, Abovsky M, Hauschild AC, Tsay M, et al. mirDIP 4.1-integrative database of human microRNA target predictions. Nucleic Acids Res. 2018;46(D1):D360–70. PubMed https://doi.org/10.1093/nar/gkx1144

17. Andrés-León E, González Peña D, Gómez-López G, Pisano DG. miRGate: a curated database of human, mouse and rat miRNA-mRNA targets. Database. 2015;2015:bav035.

18. Chou CH, Shrestha S, Yang CD, Chang NW, Lin YL, Liao KW, et al. miRTarBase update 2018: a resource for experimentally validated microRNA-target interactions. Nucleic Acids Res. 2018;46(D1):D296–302. PubMed https://doi.org/10.1093/nar/gkx1067

19. Benjamini Y, Drai D, Elmer G, Kafkafi N, Golani I. Controlling the false discovery rate in behavior genetics research. Behav Brain Res. 2001;125:279–84. PubMed https://doi.org/10.1016/S0166-4328(01)00297-2

Appendix Table 1. Characteristics of microRNAs detected in cell-conditioned media of human brain endothelial cells exposed to *Plasmodium falciparum*−infected and noninfected erythrocytes*

Sample	Total reads	Quality filtered	Complexity filtered	Length filtered	Good-quality reads	rRNA	tRNA	miRNA	Unknown	Different miRNAs
t1_niE-1	4,681,945	4	34	337,979	4,343,966	270,763	2,366,921	75,187	1,631,095	231
t1_niE-2	11,411,515	62	1,191	562,570	10,848,945	3,679,590	1,543,964	252,710	5,372,681	212
t1_niE-3	10,012,344	72	1,507	1,000,811	9,011,533	3,064,588	1,243,462	449,402	4,254,081	465
t1_3D7-iE-1	12,991,483	2	81	850,480	12,141,003	912,334	5,575,569	369,947	5,283,153	233
t1_3D7-iE-2	6,470,351	0	4	225,621	6,244,730	281,177	3,677,882	46,237	2,239,434	240
t1_3D7-iE-3	11,824,305	42	1,023	965,079	10,859,226	3,529,982	1,986,414	392,862	4,949,968	256
t1_ePCR-iE-1	5,571,743	8	60	1450,083	4,121,660	585,628	982,702	215,775	2,337,555	363
t1_ePCR-iE-2	2,366,064	12	215	591,454	1,774,610	434,035	137,804	201,756	1,001,015	122
t1_ePCR-iE-3	6,400,097	12	387	651,993	5,748,104	1,148,564	1,398,684	322,921	2,877,935	181
t24_niE-1	33,411,780	61	1,326	5,688,689	27,723,091	5,762,892	4,456,704	2,896,826	14,606,669	157
t24_niE-2	10,227,177	37	881	899,113	9,328,064	3,242,238	1,646,379	328,760	4,110,687	137
t24_niE-3	6,482,702	2	28	203,593	6,279,109	347,523	4,011,059	58,043	1,862,484	119
t24_3D7-iE-1	14,668,936	0	51	730,638	13,938,298	663,276	7,270,210	498,805	5,506,007	101
t24_3D7-iE-2	5,034,919	0	7	156,980	4,877,939	208,106	2,998,788	161,344	1,509,701	146
t24_3D7-iE-3	57,868,438	377	9,448	5,474,274	52,394,164	16,356,374	8,786,690	2,280,235	24,970,865	230
t24_ePCR-iE-1	6,163,503	2	2	156,258	6,007,245	212,547	3,808,455	92,989	1,893,254	120
t24_ePCR-iE-2	7,262,082	46	916	1,057,919	6,204,163	2,149,075	896,948	293,743	2,864,397	223
t24_ePCR-iE-3	4,907,246	1	6	246,902	4,660,344	336,305	2,697,248	52,875	1,573,916	118

*ePCR-iE, cytoadherent *P. falciparum*−infected erythrocyte; miRNA, microRNA; niE, noninfected erythrocyte, 3D7-iE, non-cytoadherent *P. falciparum*−infected erythrocyte

Appendix Table 2. MicroRNAs differentially expressed in cell-conditioned media of human brain endothelial cells were exposed to niE and compared with ePCR-iE after 1 h incubation*

miRNA	baseMean	log2FoldChange	padj
hsa-miR-150–5p	3628.925319	7.106957417	2.20E-64
hsa-miR-1246	8513.8312	6.854156394	2.86E-33
hsa-miR-342–3p	663.0033186	5.589928255	2.74E-29
hsa-miR-1290	958.7797602	6.480019242	3.64E-22
hsa-miR-223–5p	359.7886812	6.949480635	3.31E-19
hsa-miR-4791	186.7263031	9.764657543	1.81E-13
hsa-miR-143–3p	3014.137094	4.242367581	1.25E-12
hsa-miR-3690	70.86488948	9.209904393	3.00E-12
hsa-miR-145–5p	72.96215294	8.781293485	4.09E-12
hsa-miR-146b-5p	572.9246948	3.850563079	7.29E-12
hsa-miR-150–3p	60.38153154	7.465472022	4.02E-11
hsa-miR-223–3p	3583.963415	10.60183039	6.42E-11
hsa-miR-197–3p	1848.704656	3.167278515	3.68E-10
hsa-miR-6842–3p	121.4605639	5.034134649	3.78E-10
hsa-miR-27a-5p	159.1304428	4.762393491	2.69E-09
hsa-miR-4286	28.41313356	7.592582565	5.34E-09
hsa-miR-4732–3p	39.85412873	−4.344998011	9.19E-09
hsa-miR-29a-3p	264.2422459	3.036225197	3.23E-08
hsa-miR-23a-3p	1648.646951	2.426234941	5.38E-08
hsa-miR-1291	24.09578022	7.678294911	6.73E-08
hsa-miR-140–3p	6094.803676	2.906210556	8.46E-08
hsa-miR-361–3p	518.887784	2.698437793	9.62E-08
hsa-miR-582–3p	45.25401955	5.187956688	1.71E-07
hsa-miR-363–3p	412.4688189	−2.480558611	2.01E-07
hsa-miR-1299	102.3727512	4.139411714	2.36E-07
hsa-miR-28–3p	1018.820725	2.668213456	5.27E-07
hsa-miR-766–3p	29.59956282	5.9824388	5.65E-07
hsa-miR-146b-3p	27.95214056	5.751583995	6.65E-07
hsa-miR-146a-5p	395.7308176	2.295825898	1.07E-06
hsa-miR-486–3p	442.3385331	−2.280314186	1.42E-06
hsa-miR-199b-5p	17.69789788	6.652284259	1.65E-06
hsa-miR-148a-3p	21311.06097	2.967223068	2.14E-06
hsa-miR-769–5p	78.16221633	3.454304058	2.22E-06
hsa-miR-132–3p	13.41828033	6.795969882	2.82E-06
hsa-miR-504–5p	16.20689324	6.842868855	3.34E-06
hsa-miR-92a-3p	65823.55513	−2.10142854	4.67E-06
hsa-miR-330–3p	53.90163431	3.129351136	4.88E-06
hsa-miR-200c-3p	15.27230031	6.716323846	5.34E-06
hsa-miR-486–5p	186570.8541	−2.309900141	5.98E-06
hsa-miR-338–5p	16.14897785	6.511596255	7.43E-06
hsa-miR-183–5p	757.7566497	−2.260214693	7.87E-06
hsa-miR-1273h-5p	11.10031853	6.530890359	1.55E-05
hsa-miR-342–5p	93.52411001	3.308610483	4.55E-05
hsa-miR-326	16.42493563	5.988631002	4.61E-05
hsa-miR-618	15.6716072	6.016209684	4.61E-05
hsa-miR-191–5p	2983.128623	1.523838499	7.79E-05
hsa-miR-27a-3p	285.7014296	1.731405393	7.96E-05
hsa-miR-328–3p	271.7224572	2.170776673	7.96E-05
hsa-miR-451a	14357.385	−1.83622576	9.53E-05
hsa-miR-3614–5p	9.33818402	6.123669779	0.000106957
hsa-miR-30e-3p	815.6225605	2.360699038	0.000125365
hsa-miR-3653–3p	9.955406247	6.067314627	0.000138372
hsa-miR-16–2-3p	99.02227371	−2.704373872	0.000138392
hsa-miR-941	1372.569505	2.112993367	0.000155687
hsa-miR-1273h-3p	8.79013326	5.927996135	0.000189765
hsa-miR-584–5p	74.74437394	−2.177917358	0.000200673
hsa-miR-361–5p	31.19551383	3.652271942	0.000286557
hsa-miR-423–5p	9496.481452	−1.666500408	0.000301254
hsa-miR-425–3p	23.4899942	3.50770916	0.000497233
hsa-miR-1908–5p	5.40110314	−6.802848578	0.000500329
hsa-miR-144–5p	73.08790204	−2.608545423	0.000614181
hsa-miR-148a-5p	7.077958372	5.991607608	0.000826919
hsa-miR-3960	21.82641113	−3.014306593	0.000826919
hsa-miR-92b-3p	284.8388438	−1.662148164	0.001252142
hsa-miR-625–3p	147.3917485	2.170185206	0.001322084
hsa-miR-320a	3462.527306	−1.402939938	0.00139293
hsa-miR-345–5p	22.71828536	3.943197206	0.00139293

miRNA	baseMean	log2FoldChange	padj
hsa-miR-1260a	50.29735001	2.615764632	0.001605391
hsa-let-7i-5p	12361.71358	−1.302787012	0.001797592
hsa-miR-619–5p	22.7811983	3.161529188	0.001900387
hsa-miR-122–5p	3385.264898	−1.584467253	0.002340813
hsa-miR-340–5p	46.36446243	2.653771404	0.002544196
hsa-miR-144–3p	48.36612972	−2.49500384	0.002555714
hsa-miR-126–3p	892.3500608	−1.625098791	0.003126621
hsa-miR-543	129.0107724	−1.784228026	0.003228785
hsa-miR-22–3p	198.2421283	−1.339256703	0.003317174
hsa-miR-125a-3p	25.61739412	2.647707932	0.0045958
hsa-miR-26b-3p	5.972701619	5.518835758	0.0045958
hsa-miR-365a-3p	53.55418216	2.123552072	0.0045958
hsa-miR-365b-3p	53.55418216	2.123552072	0.0045958
hsa-let-7b-5p	11122.0756	−1.385051301	0.004654547
hsa-miR-340–3p	9.403598252	4.19564441	0.007285806
hsa-miR-3158–3p	143.2936325	−1.487178473	0.00782372
hsa-miR-3909	4.660678895	5.033669701	0.008065745
hsa-miR-505–3p	19.61789922	3.184975964	0.009512761
hsa-miR-96–5p	6.228218724	−4.412956083	0.01160297
hsa-miR-92b-5p	15.60484631	−2.774216268	0.011905118
hsa-miR-5684	14.25740308	−3.392469579	0.012209695
hsa-miR-574–3p	151.5421548	1.44861864	0.012209695
hsa-miR-589–5p	112.3400591	1.712421621	0.012209695
hsa-miR-1260b	38.28153971	1.901084012	0.015737938
hsa-miR-17–5p	56.12478112	−1.912960954	0.017636633
hsa-miR-4488	301.3772581	1.686545438	0.019800269
hsa-miR-3150b-3p	9.284489454	3.713227496	0.020280067
hsa-miR-503–5p	39.07218516	−1.757011842	0.023641179
hsa-miR-24–2-5p	7.48136792	3.967586096	0.024393346
hsa-miR-4508	74.78488456	−1.444481229	0.024393346
hsa-miR-193a-5p	184.9649622	1.410027061	0.025848637
hsa-miR-148b-3p	604.5039106	1.071494091	0.025962098
hsa-miR-24–3p	347.9262528	1.011562924	0.025962098
hsa-miR-26a-5p	2978.105792	1.023914595	0.030449772
hsa-let-7a-5p	14867.14987	−1.186931323	0.032443997
hsa-miR-1180–3p	56.26387509	−1.461006415	0.032443997
hsa-miR-128–3p	443.1001246	1.234565868	0.032663654
hsa-miR-4732–5p	37.4933489	−1.669985619	0.033292936
hsa-miR-194–5p	19.87025713	−2.652995088	0.035040129
hsa-let-7e-5p	315.5665007	−1.266168593	0.036277702
hsa-miR-222–3p	1950.75622	−1.054430196	0.04170154
hsa-miR-744–5p	307.4576353	1.101237911	0.04395607
hsa-miR-93–3p	19.14613315	2.21447109	0.04444623
hsa-miR-28–5p	15.19613321	2.619036378	0.047605172

*Positive FoldChange indicates overexpression in niE. RNA sequencing data included a total of 363 microRNAs. baseMean is the mean normalized expression of the miRNAs in all the samples; padj is adjusted for multiple testing by the Benjamini-Hochberg method. ePCR-iE, adherent FCR3 expression endothelial receptor of protein C-infected erythrocytes; miRNA, microRNA; niE, noninfected erythrocytes.

Appendix Table 3. MicroRNAs differentially expressed in cell-conditioned media of human brain endothelial cells when exposed to 3D7-iE and compared with ePCR-iE after 1 h incubation*

miRNA	baseMean	log2FoldChange	padj
hsa-miR-150–5p	3628.925319	6.447621237	8.41E-53
hsa-miR-1246	8513.8312	6.448500505	2.44E-29
hsa-miR-342–3p	663.0033186	4.805719054	1.87E-21
hsa-miR-1290	958.7797602	6.047501723	3.28E-19
hsa-miR-143–3p	3014.137094	4.973540967	3.06E-17
hsa-miR-223–5p	359.7886812	6.431781207	1.96E-16
hsa-miR-4791	186.7263031	10.38932761	2.56E-15
hsa-miR-145–5p	72.96215294	8.773111499	5.33E-12
hsa-miR-23a-3p	1648.646951	2.919080408	3.83E-11
hsa-miR-146b-5p	572.9246948	3.720217317	5.04E-11
hsa-miR-423–5p	9496.481452	−2.845800299	1.45E-10
hsa-miR-223–3p	3583.963415	10.40824443	1.77E-10
hsa-miR-28–3p	1018.820725	3.236927509	7.54E-10
hsa-miR-197–3p	1848.704656	3.045327492	2.21E-09
hsa-miR-3690	70.86488948	7.939040485	3.04E-09
hsa-miR-363–3p	412.4688189	−2.752949366	7.66E-09
hsa-miR-451a	14357.385	−2.623190083	1.10E-08
hsa-miR-4732–3p	39.85412873	−4.446862598	1.22E-08
hsa-miR-4286	28.41313356	7.221117949	3.72E-08
hsa-miR-1299	102.3727512	4.296659106	8.94E-08
hsa-miR-146a-5p	395.7308176	2.416585365	3.27E-07
hsa-miR-320a	3462.527306	−2.147110555	4.06E-07
hsa-miR-150–3p	60.38153154	5.841471731	5.53E-07
hsa-miR-3158–3p	143.2936325	−2.671376413	6.03E-07
hsa-miR-199b-5p	17.69789788	6.756414418	1.41E-06
hsa-miR-326	16.42493563	7.000378659	1.46E-06
hsa-miR-92a-3p	65823.55513	−2.206241834	1.80E-06
hsa-miR-618	15.6716072	6.882512894	2.54E-06
hsa-miR-27a-5p	159.1304428	3.853017578	2.61E-06
hsa-miR-361–5p	31.19551383	4.557109213	3.99E-06
hsa-miR-766–3p	29.59956282	5.475435141	7.17E-06
hsa-miR-338–5p	16.14897785	6.585842515	7.56E-06
hsa-miR-1180–3p	56.26387509	−2.865258808	1.15E-05
hsa-miR-486–5p	186570.8541	−2.257048303	1.36E-05
hsa-miR-486–3p	442.3385331	−2.077360932	1.53E-05
hsa-miR-140–3p	6094.803676	2.378049094	1.89E-05
hsa-miR-1291	24.09578022	6.218174392	2.27E-05
hsa-miR-29a-3p	264.2422459	2.377846317	2.52E-05
hsa-miR-4508	74.78488456	−2.537316275	3.24E-05
hsa-miR-6842–3p	121.4605639	3.458457239	3.89E-05
hsa-miR-200c-3p	15.27230031	6.110652566	5.42E-05
hsa-miR-504–5p	16.20689324	6.10449083	5.42E-05
hsa-miR-582–3p	45.25401955	4.02067135	9.79E-05
hsa-miR-769–5p	78.16221633	2.900427661	0.000122239
hsa-miR-27a-3p	285.7014296	1.699710038	0.000132728
hsa-miR-625–3p	147.3917485	2.549490741	0.000132728
hsa-miR-340–5p	46.36446243	3.201883577	0.000214609
hsa-miR-144–3p	48.36612972	−3.0660485	0.000220627
hsa-miR-425–3p	23.4899942	3.724522238	0.000223367
hsa-miR-320b	420.1406035	−1.862313231	0.000276457
hsa-miR-365a-3p	53.55418216	2.655154806	0.000276457
hsa-miR-365b-3p	53.55418216	2.655154806	0.000276457
hsa-miR-132–3p	13.41828033	5.410556286	0.000380553
hsa-let-7i-5p	12361.71358	−1.47141141	0.000382512
hsa-miR-30e-3p	815.6225605	2.226049992	0.000382512
hsa-miR-92b-5p	15.60484631	−4.163857943	0.0003924
hsa-miR-146b-3p	27.95214056	4.242630881	0.000482005
hsa-miR-92b-3p	284.8388438	−1.785328471	0.000505668
hsa-miR-4732–5p	37.4933489	−2.594968757	0.000643162
hsa-miR-122–5p	3385.264898	−1.753744088	0.000704175
hsa-miR-144–5p	73.08790204	−2.558810315	0.00089817
hsa-miR-3653–3p	9.955406247	5.420823215	0.000963768
hsa-miR-330–3p	53.90163431	2.33736255	0.001216256
hsa-miR-2115–3p	6.041154984	5.834660721	0.001395787
hsa-miR-183–5p	757.7566497	−1.689145385	0.001439809
hsa-miR-1273h-3p	8.79013326	5.232979185	0.001476418
hsa-miR-1273h-5p	11.10031853	5.004655278	0.001837808

miRNA	baseMean	log2FoldChange	padj
hsa-miR-345–5p	22.71828536	3.888926893	0.001844401
hsa-miR-361–3p	518.887784	1.669833063	0.001878529
hsa-miR-328–3p	271.7224572	1.756856697	0.002066471
hsa-miR-3614–5p	9.33818402	5.084630328	0.002066471
hsa-miR-1260a	50.29735001	2.56883531	0.002146885
hsa-miR-191–5p	2983.128623	1.22004135	0.002216696
hsa-miR-505–3p	19.61789922	3.426913927	0.00528521
hsa-miR-4685–3p	26.37952361	−2.212963535	0.006078573
hsa-miR-148a-3p	21311.06097	1.831991607	0.006860876
hsa-miR-126–3p	892.3500608	−1.523444179	0.007103832
hsa-let-7b-5p	11122.0756	−1.342668008	0.007601427
hsa-miR-5189–5p	4.690302347	−6.195706422	0.008125759
hsa-miR-4448	19.99074734	2.751420355	0.008596921
hsa-miR-28–5p	15.19613321	3.329553964	0.008697526
hsa-miR-619–5p	22.7811983	2.721712643	0.011008689
hsa-miR-5684	14.25740308	−3.504790338	0.011818106
hsa-miR-221–5p	30.33237403	2.235527273	0.014993794
hsa-miR-24–3p	347.9262528	1.101827408	0.015833224
hsa-miR-193a-5p	184.9649622	1.491052572	0.020130112
hsa-miR-22–3p	198.2421283	−1.113442482	0.021327869
hsa-miR-93–3p	19.14613315	2.52716344	0.021327869
hsa-miR-2110	117.7715931	−1.410612056	0.02301949
hsa-miR-342–5p	93.52411001	2.045398548	0.02301949
hsa-miR-543	129.0107724	−1.437070341	0.02604373
hsa-miR-155–5p	135.1369051	1.308204109	0.02781228
hsa-miR-152–3p	52.85362465	1.703147513	0.027818637
hsa-miR-584–5p	74.74437394	−1.39071939	0.029085671
hsa-let-7e-5p	315.5665007	−1.333615616	0.030343738
hsa-miR-1908–5p	5.40110314	−4.518938959	0.033717462
hsa-miR-16–2-3p	99.02227371	−1.642902948	0.035497673
hsa-miR-3150b-3p	9.284489454	3.477104992	0.037288673
hsa-miR-106a-5p	5.273323169	−4.723234986	0.046728731
hsa-miR-15b-5p	59.88239009	1.339085366	0.048947187

*Positive FoldChange indicates overexpression in 3D7-iE. RNA sequencing data included a total of 363 microRNAs. baseMean is the mean normalized expression of the miRNAs in all the samples. padj is adjusted for multiple testing by the Benjamini-Hochberg method. ePCR-iE, adherent FCR3 expression endothelial receptor of protein C-infected erythrocytes; 3D7-iE, nonadherent 3D7-infected erythrocytes; miRNA, microRNA.

Appendix Table 4: Characteristics of different miRNAs detected in plasma from children recruited in 2006 for study of malaria, Mozambique

Sample	Total reads	Quality filtered	Complexity filtered	Length filtered	Good-quality reads	rRNA	tRNA	miRNA	Unknown	Different miRNAs
525881.3	10,711,219	1,019	496	3,093,606	7,617,613	1,831,500	651,064	807,691	4,327,358	395
525884.4	13,220,660	803	702	3,888,349	9,332,311	966,969	596,303	1,966,978	5,802,061	574
525885.1	10,702,582	333	566	2,319,260	8,383,322	1,232,736	1,136,730	1,751,858	4,261,998	548
525887.5	13,661,026	1,124	53	2,762,425	10,898,601	594,196	670,121	2,832,128	6,802,156	539
525889.9	26,426,632	1,073	384	2,617,714	23,808,918	2,136,483	2,578,042	5,776,301	13,318,092	642
525891.2	9,524,995	439	270	1,873,951	7,651,044	699,777	854,636	1,589,401	4,507,230	593
525893.6	2,162,887	79	49	120,186	2,042,701	127,077	390,794	440,733	1,084,097	331
525896.7	17,421,782	1,908	548	5,626,333	11,795,449	1,265,772	839,750	1,030,609	8,659,318	341
525898.1	3,320,188	126	118	832,015	2,488,173	142,443	215,289	1,663,216	467,225	425
525899.8	5,937,637	296	454	1,500,015	4,437,622	572,501	700,764	517,740	2,646,617	355
525903.0	14,957,552	2	276	2,265,449	12,692,103	1,692,270	3,040,674	1,194,073	6,765,086	292
525909.2	10,293,853	2,128	224	3,483,287	6,810,566	768,196	599,146	1,269,410	4,173,814	412
544393.2	9,208,074	489	675	2,151,722	7,056,352	1,496,125	1,507,206	883,567	3,169,454	501
544394.9	13,598,660	996	1,250	6,286,338	7,312,322	1,247,933	758,898	1,477,246	3,828,245	580
544395.6	16,309,029	973	1,449	3,847,507	12,461,522	3,653,367	2,378,483	1,189,219	5,240,453	554
544396.3	7,566,573	276	197	1,246,660	6,319,913	627,080	1,632,315	1,027,308	3,033,210	531
544397.0	6,772,398	266	222	1,805,393	4,967,005	1,187,429	550,612	675,373	2,553,591	498
544404.3	7,386,300	287	354	1,583,391	5,802,909	433,117	555,473	996,837	3,817,482	581
544406.7	575,642	0	1	94,684	480,958	34,498	156,803	223,001	66,656	337
544407.4	322,699	7	47	33,711	288,988	26,869	86,815	40,357	134,947	199
544408.1	10,702,037	390	839	2,161,657	8,540,380	710,955	2,179,306	1,632,353	4,017,766	663
544413.5	15,424,105	20	208	3,016,358	12,407,747	2,130,079	1,918,422	2,473,544	5,885,702	276
544415.9	15,845,270	581	585	4,355,557	11,489,713	1,693,453	1,942,665	3,572,255	4,281,340	757
544417.3	320,995	6	47	49,727	271,268	79,124	58,275	53,365	80,504	229
544423.4	12,314,227	511	879	2,620,234	9,693,993	1,146,777	1,257,197	2,022,726	5,267,293	620
544425.8	6,960,365	244	319	1,393,757	5,566,608	452,538	1,278,619	967,048	2,868,403	473
544430.2	29,411,214	2,681	2,251	7,142,325	22,268,889	2,330,062	2,430,827	2,447,114	15,060,886	472
544431.9	12,913,067	374	771	2,019,212	10,893,855	1,553,982	2,423,487	2,087,189	4,829,197	298
544434.0	9,194,679	428	55	1,158,428	8,036,251	1,187,467	1,514,795	1,642,793	3,691,196	433
544436.4	10,628,594	352	242	1,424,162	9,204,432	1,030,961	2,030,035	2,940,439	3,202,997	465
544439.5	18,820,329	990	1,239	3,728,346	15,091,983	1,867,354	2,041,446	1,558,034	9,625,149	659
566322.2	9,096,499	219	526	970,253	8,126,246	222,824	1,176,992	1,440,172	5,286,258	463
566323.9	12,512,273	307	147	681,565	11,830,708	373,103	468,855	8,338,268	2,650,482	770
566324.6	7,407,842	271	230	799,057	6,608,785	473,056	1,541,802	2,306,487	2,287,440	644
566327.7	265,530	5	26	39,505	226,025	23,941	18,230	86,877	96,977	274
566329.1	575,470	7	48	23,763	551,707	59,080	163,228	133,556	195,843	311
566332.1	9,282,984	18	577	2,188,722	7,094,262	936,251	1,105,618	2,509,571	2,542,822	310
566333.8	12,485,750	1,976	85	3,068,214	9,417,536	1,147,898	945,883	1,973,103	5,350,652	669
566335.2	16,300,010	1,628	450	3,430,462	12,869,548	1,439,268	1,267,034	2,367,655	7,795,591	395
566353.6	12,299,560	520	92	4,379,950	7,919,610	1,052,903	619,183	2,088,724	4,158,800	612
566355.0	2,968,770	0	79	1,009,357	1,959,413	328,104	255,943	440,443	934,923	160
566356.7	15,403,237	1,053	1,726	3,642,172	11,761,065	2,419,028	1,509,598	1,633,826	6,198,613	628
566358.1	8,943,757	529	1,773	3,677,028	5,266,729	1,105,767	487,154	1,637,474	2,036,334	454
566359.8	744,578	20	72	58,563	686,015	35,746	148,801	79,994	421,474	288
566361.1	25,792,113	905	1,378	7,723,560	18,068,553	1,793,988	2,690,970	6,727,806	6,855,789	580
566362.8	12,021,808	747	24	3,274,341	8,747,467	1,603,501	18,940	10,773	7,114,253	151

Sample	Total reads	Quality filtered	Complexity filtered	Length filtered	Good-quality reads	rRNA	tRNA	miRNA	Unknown	Different miRNAs
566364.2	1,631,464	99	104	556,133	1,075,331	112,855	251,575	48,887	662,014	274
566365.9	19,129,131	2	966	1,691,465	17,437,666	2,087,295	2,389,159	7,624,018	5,337,194	399
566366.6	19,623,668	7,037	92	5,322,923	14,300,745	1,231,391	1,029,852	1,170,845	10,868,657	467
566367.3	384,010	3	32	97,518	286,492	22,854	26,912	122,521	114,205	150
579033.4	12,761,288	299	223	2,438,291	10,322,997	4,471,210	1,076,958	2,371,736	2,403,093	570
579034.1	7,225,891	25	2,766	472,409	6,753,482	472,354	2,250,034	994,990	3,036,104	465
579035.8	9,863,866	517	265	2,370,430	7,493,436	441,043	567,999	1,734,312	4,750,082	481
579036.5	145,356	0	13	17,436	127,920	4,210	11,235	65,688	46,787	196
579037.2	1,493,408	4	82	338,939	1,154,469	47,182	229,698	521,844	355,745	394
579038.9	10,367,363	371	1,380	3,078,000	7,289,363	639,425	1,550,985	991,123	4,107,830	388
579040.2	9,253,151	13	408	2,472,738	6,780,413	384,449	1,761,922	500,022	4,134,020	373
579042.6	15,960,129	470	202	2,728,371	13,231,758	638,919	3,805,700	1,934,893	6,852,246	380
579043.3	7,089,342	206	697	1,602,007	5,487,335	964,876	1,536,510	687,802	2,298,147	260
579045.7	1,982,823	2	433	143,776	1,839,047	233,623	490,286	324,346	790,792	305
579046.4	1,873,456	486	38	806,002	1,067,454	75,715	60,748	43,809	887,182	185
579048.8	1,082,686	1	50	185,056	897,630	54,474	153,016	135,343	554,797	200
579050.1	170,063	2	27	17,739	152,324	17,255	29,933	23,521	81,615	160
579051.8	10,722,970	448	158	1,708,024	9,014,946	642,574	2,552,986	1,186,376	4,633,010	313
579052.5	4,228,866	219	748	2,256,487	1,972,379	246,201	433,194	222,570	1,070,414	146
579053.2	8,618,384	462	1,092	1,949,499	6,668,885	354,969	1,025,766	994,142	4,294,008	319
579054.9	9,386,303	689	143	4,284,092	5,102,211	340,702	1,452,895	638,954	2,669,660	242
579055.6	3,526,717	94	80	1,769,909	1,756,808	199,283	294,707	223,967	1,038,851	123
579058.7	1,792,508	133	10	997,704	794,804	53,040	154,970	126,036	460,758	242
579059.4	17,665,599	8	910	1,746,197	15,919,402	1,020,271	3,146,816	6,390,514	5,361,801	365
579060.0	11,438,101	537	771	3,749,312	7,688,789	618,651	1,015,852	548,212	5,506,074	300
579061.7	33,311,564	3	1,496	2,576,915	30,734,649	2,601,559	5,795,176	11,096,716	11,241,198	442
579062.4	3,451,830	156	2	1,762,819	1,689,011	202,947	239,684	218,299	1,028,081	116
579064.8	8,994,815	278	178	2,721,236	6,273,579	840,779	987,181	593,171	3,852,448	161
579065.5	18,176,617	7,425	386	6,172,449	12,004,168	781,469	399,288	948,897	9,874,514	334
579068.6	5,128,830	305	256	1,520,699	3,608,131	300,353	890,927	810,364	1,606,487	396
579069.3	45,577,321	37	4,302	2,027,990	43,549,331	926,339	3,622,063	5,903,995	33,096,934	786
579075.4	9,827,499	20	816	1,032,689	8,794,810	363,164	665,426	1,692,835	6,073,385	515
579077.8	10,214,090	344	167	1,093,407	9,120,683	1,504,424	989,870	3,501,517	3,124,872	595
579078.5	4,481,826	234	167	2,157,860	2,323,966	119,110	242,195	281,184	1,681,477	175
579079.2	1,225,321	30	72	869,796	355,525	78,232	60,481	95,781	121,031	204
598991.0	8,703,408	2	406	1,378,261	7,325,147	439,304	989,308	2,636,136	3,260,399	546
598993.4	7,424,401	473	236	3,018,779	4,405,622	292,897	603,224	430,567	3,078,934	181

Appendix Table 5. Spearman correlations between ELISA-based HRP2 levels and miRNA relative expression levels (RELs) in plasma samples from children with malaria, Mozambique*

miRNA	rho	p value
Children recruited in 2006		
hsa-miR-10b-5p	0.415	0.020
hsa-miR-378a-3p	0.422	0.020
hsa-miR-4497	0.533	<0.001
Children recruited in 2014		
hsa-miR-122–5p	−0.016	0.892
hsa-miR-320a	0.121	0.320
hsa-miR-1246	0.066	0.588
hsa-miR-1290	0.123	0.310
hsa-miR-3158–3p	0.511	<0.001
hsa-miR-4497	0.401	<0.001

*HRP2 levels and miRNA RELs were log transformed.

Appendix Table 6. PCR efficiencies of each microRNA used for reverse transcription quantitative PCR analysis of specimens from children with malaria, Mozambique

miRNA	PCR efficiency, %
hsa-miR-122–5p	99.6
hsa-miR-320a	95.3
hsa-miR-1246	98.4
hsa-miR-1290	103.0
hsa-miR-3158–3p	91.2
hsa-miR-4497	96.9
has-miR-191–5p	93.1
hsa-miR-30d-5p	103.8
hsa-miR-148a-3p	100.1
ath-miR-159a	103.2

Appendix Table 7. Predicted targets of the 2 miRNAs (hsa-miR-3158–3p and hsa-miR-4497) validated in children with malaria recruited in 2014, Mozambique

hsa-miR-3158–3p	hsa-miR-4497
TXNIP	RUNX1
MAP3K9	CCNF
ZNF704	CTTN
BCL7A	FANCA
GATAD2B	NAB2
FBXO46	NF2
CHST15	NPY4R
FTSJ3	PRPS1
CTC1	TPM3
CABP7	UGT8
C20orf194	VEGFA
IGSF8	RAB9A
KLHL15	IRX5
NLK	BPNT1
NFASC	APPBP2
LMNB2	CD226
MRPS18A	ATP1B4
LSM4	SH2B1
XRCC6	SDF4
ZBTB39	MTPAP
UBXN2B	TMEM33
RRP7A	DUSP22
ZCCHC14	RAB22A
RANGAP1	TBC1D24
RAB3IP	ZNF490
AMOTL2	DSN1
ANXA11	FBXL18
C22orf39	LRRC27
DDX6	ATP13A4
CDK2	HIST1H2AH
ENAH	SP140L
ENPP5	CAMK2N2

hsa-miR-3158–3p	hsa-miR-4497
GDAP1L1	C10orf71
FOSL1	FAM83C
MMP15	MIPOL1
PLAG1	CCNY
NANOS1	PRPS1L1
MRTO4	CBARP
PIGQ	LHFPL3
NLGN1	C8orf82
RTN2	PPAN-P2RY11
SAMD4A	CASTOR2
PTPRJ	
THSD7A	
ZNF281	

Appendix Table 8. Clustering results of DAVID analysis in study of malaria in children, Mozambique

Category	Term	Genes	Fold enrichment	p value	Benjamini p value
GOTERM_CC_DIRECT	GO:0030687~preribosome, large subunit precursor	PPAN-P2RY11, FTSJ3, MRTO4	25.136	0.006	0.647
GOTERM_BP_DIRECT	GO:0007399~nervous system development	IGSF8, NAB2, VEGFA, NLGN1, BPNT1, PRPS1	4.875	0.007	0.977
GOTERM_CC_DIRECT	GO:0045335~phagocytic vesicle	RAB9A, ANXA11, RAB22A	18.691	0.011	0.605
GOTERM_MF_DIRECT	GO:0000287~magnesium ion binding	NLK, MTPAP, PRPS1L1, BPNT1, PRPS1	5.237	0.015	0.902
GOTERM_BP_DIRECT	GO:0006015~5-phosphoribose 1-diphosphate biosynthetic process	PRPS1L1, PRPS1	116.611	0.017	0.987
GOTERM_BP_DIRECT	GO:0002175~protein localization to paranode region of axon	NFASC, UGT8	116.611	0.017	0.987
GOTERM_BP_DIRECT	GO:0009156~ribonucleoside monophosphate biosynthetic process	PRPS1L1, PRPS1	93.288	0.021	0.974
GOTERM_MF_DIRECT	GO:0004749~ribose phosphate diphosphokinase activity	PRPS1L1, PRPS1	85.473	0.023	0.702
GOTERM_CC_DIRECT	GO:0043231~intracellular membrane-bounded organelle	CTTN, MTPAP, RANGAP1, UGT8, RUNX1, ATP13A4, DDX6	3.048	0.026	0.667
GOTERM_MF_DIRECT	GO:0019003~GDP binding	RAB9A, RAB22A, PRPS1	11.871	0.026	0.641
GOTERM_CC_DIRECT	GO:0005635~nuclear envelope	TMEM33, ANXA11, ATP1B4, RANGAP1	6.112	0.027	0.602
GOTERM_CC_DIRECT	GO:0030027~lamellipodium	ENAH, CTTN, NF2, RAB3IP	6.074	0.027	0.542
GOTERM_BP_DIRECT	GO:0030913~paranodal junction assembly	NFASC, UGT8	66.634	0.029	0.978
GOTERM_CC_DIRECT	GO:0000932~cytoplasmic mRNA processing body	LSM4, SAMD4A, DDX6	9.345	0.04	0.627
GOTERM_MF_DIRECT	GO:0030371~translation repressor activity	NANOS1, SAMD4A	38.851	0.049	0.798
GOTERM_CC_DIRECT	GO:0001726~ruffle	CTTN, NF2, RAB22A	8.099	0.052	0.674
GOTERM_BP_DIRECT	GO:0000956~nuclear-transcribed mRNA catabolic process	LSM4, MRTO4	35.88	0.054	0.996
GOTERM_BP_DIRECT	GO:0033962~cytoplasmic mRNA processing body assembly	LSM4, DDX6	35.88	0.054	0.996
GOTERM_BP_DIRECT	GO:0071425~hematopoietic stem cell proliferation	RUNX1, CTC1	33.317	0.058	0.994
GOTERM_BP_DIRECT	GO:0051301~cell division	DSN1, CCNY, CCNF, ANXA11, CDK2	3.331	0.061	0.99
GOTERM_BP_DIRECT	GO:0009165~nucleotide biosynthetic process	PRPS1L1, PRPS1	31.096	0.062	0.983
GOTERM_CC_DIRECT	GO:0042470~melanosome	RAB9A, TMEM33, ANXA11	7.217	0.063	0.708

Category	Term	Genes	Fold enrichment	p value	Benjamini p value
GOTERM_CC_DIRECT	GO:0005856~cytoskeleton	ENAH, CTTN, NF2, RAB3IP, TPM3	3.274	0.064	0.673
GOTERM_MF_DIRECT	GO:0005161~platelet-derived growth factor receptor binding	PTPRJ, VEGFA	28.491	0.067	0.837
GOTERM_BP_DIRECT	GO:0050860~negative regulation of T cell receptor signaling pathway	PTPRJ, DUSP22	27.437	0.069	0.984
GOTERM_MF_DIRECT	GO:0044822~poly(A) RNA binding	PPAN-P2RY11, RRP7A, XRCC6, ANXA11, MTPAP, LSM4, FTSJ3, SAMD4A, MRTO4, DDX6	1.892	0.075	0.828
GOTERM_BP_DIRECT	GO:0009116~nucleoside metabolic process	PRPS1L1, PRPS1	24.549	0.077	0.984
GOTERM_BP_DIRECT	GO:0051894~positive regulation of focal adhesion assembly	PTPRJ, VEGFA	22.211	0.085	0.985
GOTERM_BP_DIRECT	GO:0000027~ribosomal large subunit assembly	PPAN-P2RY11, MRTO4	22.211	0.085	0.985
GOTERM_CC_DIRECT	GO:0005770~late endosome	RAB9A, RAB22A, SDF4	5.975	0.087	0.757
GOTERM_MF_DIRECT	GO:0019901~protein kinase binding	PTPRJ, FAM83C, CCNY, CAMK2N2, CD226	2.841	0.096	0.862
GOTERM_BP_DIRECT	GO:0007422~peripheral nervous system development	NFASC, UGT8	19.435	0.096	0.987

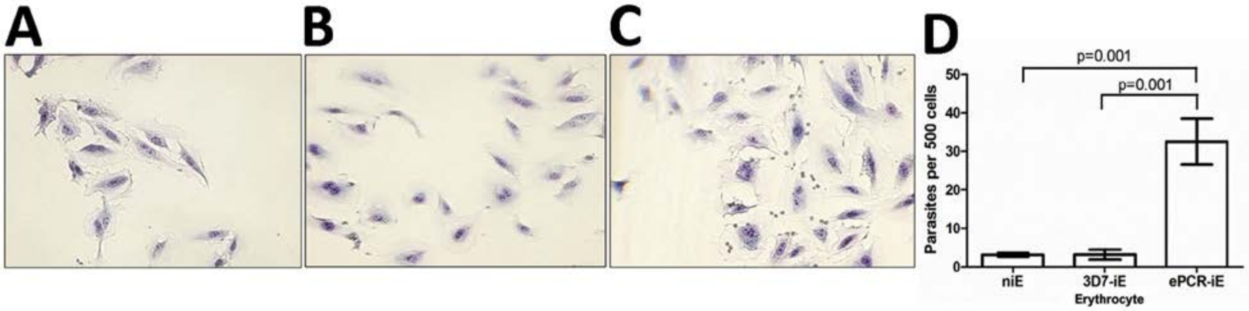

Appendix Figure 1. Cytoadhesion assay of infected erythrocytes (iEs) with human brain endothelial (HBE) cells stained with Giemsa and visualized at original magnification ×200. A) Noninfected erythrocytes (niE). B) Nonadherent 3D7-infected erythrocytes (3D7-iE). C) Adherent FCR3 expression endothelial receptor of protein C-infected erythrocytes (ePCR-iE). D) Comparison of 3 groups of infected and noninfected erythrocytes adhered to HBE cells. Bars represent mean and T lines, SD. p values were calculated by unpaired t-test.

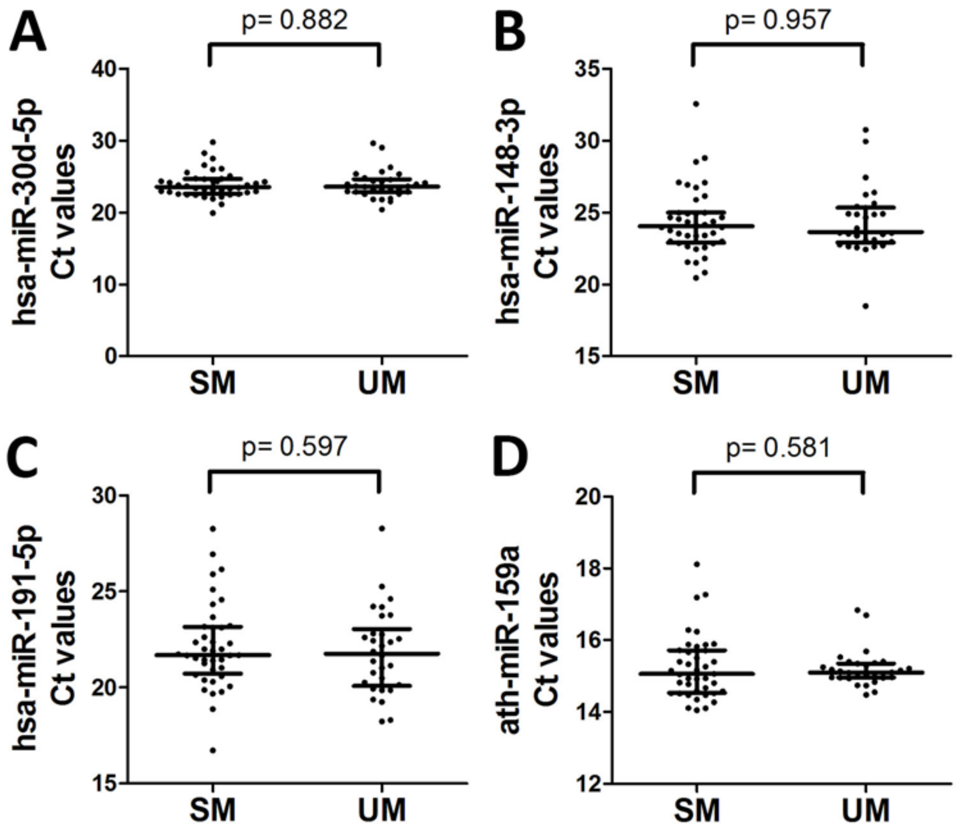

Appendix Figure 2. RT-qPCR C_t values of exogenous (ath-miR-159a) and endogenous (hsa-miR-191–5p, hsa-miR-30d-5p, and hsa-miR-148a-3p) controls in severe malaria (SM) and uncomplicated malaria (UM) groups. Distributions were compared by Mann-Whitney U test. T bars represent median and interquartile Ranges.
